# A novel single-stranded RNA virus isolated from a phytopathogenic filamentous fungus, *Rosellinia necatrix*, with similarity to hypo-like viruses

**DOI:** 10.3389/fmicb.2014.00360

**Published:** 2014-07-18

**Authors:** Rui Zhang, Shengxue Liu, Sotaro Chiba, Hideki Kondo, Satoko Kanematsu, Nobuhiro Suzuki

**Affiliations:** ^1^Group of Plant/Microbe Interactions, Institute of Plant Science and Resources, Okayama UniversityKurashiki, Okayama, Japan; ^2^Apple Research Division, National Institute of Fruit Tree Science, National Agricultural Research Organization (NARO)Morioka, Iwate, Japan

**Keywords:** novel mycovirus, *Rosellinia necatrix*, ssRNA virus, hypovirus, fusarivurs, transcriptome shotgun assembly

## Abstract

Here we report a biological and molecular characterization of a novel positive-sense RNA virus isolated from a field isolate (NW10) of a filamentous phytopathogenic fungus, the white root rot fungus that is designated as Rosellinia necatrix fusarivirus 1 (RnFV1). A recently developed technology using zinc ions allowed us to transfer RnFV1 to two mycelially incompatible *Rosellinia necatrix* strains. A biological comparison of the virus-free and -recipient isogenic fungal strains suggested that RnFV1 infects latently and thus has no potential as a virocontrol agent. The virus has an undivided positive-sense RNA genome of 6286 nucleotides excluding a poly (A) tail. The genome possesses two non-overlapping open reading frames (ORFs): a large ORF1 that encodes polypeptides with RNA replication functions and a smaller ORF2 that encodes polypeptides of unknown function. A lack of coat protein genes was suggested by the failure of virus particles from infected mycelia. No evidence was obtained by Northern analysis or classical 5′-RACE for the presence of subgenomic RNA for the downstream ORF. Sequence similarities were found in amino-acid sequence between RnFV1 putative proteins and counterparts of a previously reported mycovirus, Fusarium graminearum virus 1 (FgV1). Interestingly, several related sequences were detected by BLAST searches of independent transcriptome assembly databases one of which probably represents an entire virus genome. Phylogenetic analysis based on the conserved RNA-dependent RNA polymerase showed that RnFV1, FgV1, and these similar sequences are grouped in a cluster distinct from distantly related hypoviruses. It is proposed that a new taxonomic family termed *Fusariviridae* be created to include RnFV1 and FgV1.

## Introduction

An increasing number of mycoviruses (fungal viruses) have been reported from various host fungi (Urayama et al., 2010, 2014; Cai and Hillman, 2013; Chiba et al., 2013a,b; Jiang et al., 2013; Kalifa and Pearson, 2013; Kondo et al., 2013b; Nibert et al., 2014). In addition to conventional approaches, BLAST searches of the host transcriprome, and genome databases and subsequent molecular analyses have provided an opportunity for discovery of novel viruses and virus-like sequences (Kondo et al., 2013a). Reported mycoviruses are now classified into over 15 families (http://talk.ictvonline.org/default.aspx) including the two newly established families (*Megabirnaviridae* and *Quadriviridae*) and a recently proposed family, *Botybirnaviridae* to the International Committee on Taxonomy of Viruses (ICTV) (http://talk.ictvonline.org/default.aspx) (King et al., 2011; Wu and Li, 2013). While most of them have either double-stranded (ds) or single-stranded (ss) positive-sense (+) RNA genomes (Ghabrial and Suzuki, 2009), viruses with negative-strand (−) RNA genomes or ssDNA genomes have been reported in the past few years (Yu et al., 2010; Kondo et al., 2013a). (+)ssRNA mycoviruses are represented by members of the families *Hypoviridae, Narnaviridae, Alphaflexiviridae, Gammaflexiviridae, Endornaviridae*, and *Barnaviridae*. In addition, there are several species that have not yet been assigned to any genus or family. Those viruses include Sclerotinia sclerotiorum RNA virus L (SsRV-L) (Liu et al., 2009), Fusarium graminearum virus 1 (FgV1) (Kwon et al., 2007), Sclerophthora macrospora viruses A and B (SmVA and SmVB) (Honkura et al., 1983; Yokoi et al., 1999, 2003), and Diaporthe RNA virus (DaRV) (Moleleki et al., 2003). Based on RNA-dependent RNA polymerase (RdRp)-based dendrograms, SsRV-L and DaRV are phylogenetically related to animal and plant viruses, while FgV1 is phylogenetically related to members of the family *Hypoviridae*.

Establishment of a viral etiology in a fungal host is generally difficult and frequently encounters technical difficulties in virus elimination from a virus-carrying strain and virus introduction into a virus-free strain. Elimination of viruses from their fungal hosts may be achieved by protoplasting, hyphal tipping, or single-spore isolation with or without inhibitors of virus replication (Aoki et al., 2009; Chiba et al., 2009; Herrero and Zabalgogeazcoa, 2011; Lin et al., 2012, 2013). Whether these methods are applicable depends on the virus/host-fungus systems. For example, *Rosellinia necatrix* rarely sporulates under laboratory conditions (Nakamura et al., 2002), and some victoriviruses (dsRNA genome) are difficult to be eliminated in this fungus (Chiba et al., 2013a). Virus introduction into fungal cells may be possible by transformation of infectious cDNA clones of some viruses or transfection of synthetic viral transcripts or virions (Choi and Nuss, 1992; Chen et al., 1994; Hillman et al., 2004; Chiba et al., 2009). Protoplast fusion is an alternative to transfection that artificially introduces mycoviruses (Lee et al., 2011). Furthermore, an innovative method was recently developed by Ikeda et al. (2013) using zinc compounds that are believed to retard programmed cell death reactions upon hyphal contact between two vegetatively incompatible fungal strains of the same species causing them to anastomose leading to horizontal virus transmission between the two strains.

*R. necatrix* is an important soil-borne fungal pathogen of perennial crops and emerged recently as a fungal host for studying virus/host and virus/virus interactions (Kondo et al., 2013b). Extensive screens of Japanese field isolates for mycoviruses with potential for virocontrol (a form of biocontrol of phytopathogenic fungi using mycoviruses) (Chiba et al., 2009; Ghabrial and Suzuki, 2009) showed a relatively high (20%) incidence of virus infections in the fungus (Matsumoto, 1998; Arakawa et al., 2002; Ikeda et al., 2004). Subsequent molecular characterization showed the presence of diverse viruses isolated from this fungus belonging to at least 5 families including *Partitiviridae, Reoviridae, Totiviridae, Quadriviridae*, and *Megabirnaviridae*. All these characterized viruses of *R. necatrix* have dsRNA genomes that are either undivided (totiviruses) or divided (the rest). Importantly, of these, Rosellinia necatrix megabirnavirus 1 (RnMBV1) and mycoreovirus 3 (MyRV3) attract attention because of their potential as virocontrol agents. Furthermore, many of them were shown to be able to infect a taxonomically distinct heterologous fungus, *Cryphonectria parasitica*, in which these viruses are targeted by RNA silencing, an anti-virus host defense (Kanematsu et al., 2010; Chiba et al., 2013a,b; Salaipeth et al., 2014).

Here we report a thorough characterization of a novel (+)ssRNA virus termed Rosellinia necatrix fusarivirus 1 strain NW10 (RnFV1-NW10) that has been shown by the new zinc chloride-based method to asymptomatically infect *R. necatrix*. We propose that this virus be placed into a group together with the previously reported FgV1 for which a new family “*Fusariviridae*” is proposed. Interestingly, related sequences are found in fungal and plant transcriptome shotgun libraries (TSA), suggesting a wide prevalence of similar viruses across kingdoms.

## Materials and methods

### Fungal strains

*R. necatrix* strain NW10 (harboring N10 dsRNA) was isolated from an apple tree in Nagano, belonging to the mycelial compatibility group 442 (MCG442) (Yaegashi et al., 2013). Hygromycin B-resistant strains RT60-2, RT37-1, and RT45-1 were derived from W57-T25 (Chiba et al., 2013b), W370T1 (Kanematsu et al., 2004), and W97 (Kanematsu et al., 2004), and were used as virus recipients. RT60-2, RT37-1, and RT45-1 belong to MCG54, MCG139, and MCG80. Strains W779 and W1118 were previously described as containing RnMBV1 and Rosellinia necatrix quadrivirus 1 strain W1118 (RnQV1-W1118) (Chiba et al., 2009; Lin et al., 2013). All strains were cultured at 24°C on Difco potato dextrose agar (PDA; Becton Dickinson, Sparks, MD), Vogel's medium or in Difco potato dextrose broth (PDB) in the dark and kept at 4°C until used.

### Purification of virus particles

Fungal strain NW10 was grown for 14 days in PDB. Mycelia (approximately 30 g, wet weight) were harvested and ground to powder in the presence of liquid nitrogen. The homogenates were mixed with 30 ml of 0.1 M sodium phosphate, pH 7.0 (PB), and clarified twice with 20% (v/v) CCl_4_. Sodium chloride and polyethylene glycol 6000 were added to final concentrations of 1% and 6%, respectively. After being stirred for 2 h at 4°C, the suspension was centrifuged at 16,000 g for 20 min. Pellets were suspended in 10 ml of 0.05 M PB, and centrifuged at 7000 g for 20 min. The supernatant was re-centrifuged through a 20% sucrose cushion (3 ml) at 80,000 g for 2 h. The pellet was suspended in 1 ml 0.05 M PB, and fractionated through a 10–40% sucrose gradient by centrifugation at 80,000 g for 2 h. Fractions (1.5 ml each) with 3 ml 0.05 M PB were subjected to centrifugation again at 80,000 g for 2 h, and the pellets were suspended in 20 μl of 0.05 M PB.

### Zinc ion-mediated virus transmission

Two mycelial blocks (approximately 4 × 4 mm) from the growing edge of 5-day-old cultures of donor strain NW10, and one each of recipient strains RT60-2, RT37-1, and RT45-1, were placed on PDA with 1.0 mM ZnCl_2_ (Ikeda et al., 2013). Two blocks were placed 2–3 mm apart, approximately 5 mm from the edge of the plate. After 30 days of culture at 24°C in the dark, mycelial agar discs taken from different positions of the recipient colony were inoculated on PDA with 50 mg/ml hygromycin. Mycelial discs were sub-cultured on PDA covered with cellophane for 5–7 days and used for Northern blotting.

### RNA extraction and analyses

Total nucleic acid preparations were obtained by the method of Sun and Suzuki (2008). DsRNA was further purified by binding with CC41 cellulose (Whatman). Briefly, total nucleic acids were incubated with CC41 cellulose in STE buffer (10 mM Tris-HCl, pH 8.0, 150 mM NaCl, and 1 mM EDTA) containing 15% (v/v) ethanol. After washing the cellulose with the STE-15% ethanol for 3 times, dsRNA was eluted by STE buffer and precipitated by the addition of 2 volumes of ethanol. Recovered dsRNAs were analyzed by agarose gel electrophoresis and a gel-purified fragment was subjected to sequence analysis. ssRNA sampling and Northern blotting analysis using synthetic cDNA probes were conducted as described by Chiba et al. (2013a). *In vitro* transcription of RnFV1 positive-sense RNAs were performed following the method of Salaipeth et al. (2014), after linearization of plasmids with the *Bsa* I restriction enzyme.

### Universally primed (UP)-PCR

Genomic DNA was extracted by the CTAB (cetyl trimethyl ammonium bromide) method. To confirm genetic backgrounds of recipients, UP-PCR was performed to detect DNA polymorphisms by using the AS4 (5′-TGTGGGCGCTCGACAC-3′) and AS15 (5′-GGCTAAGCGGTCGTTAC-3′) primers (Ikeda et al., 2004). PCR products were separated by electrophoresis on a 1.2% agarose gel in 0.5× TAE buffer.

### cDNA cloning and sequencing analysis

A cDNA library was constructed by a classical, non-PCR-based method as described by Lin et al. (2012). Plasmid clones carrying cDNA inserts of 1.5–3.0 kbp were sequenced in both directions. Classical Rapid Ampliflcation of cDNA Ends (RACE) and RNA-ligase-mediated (RLM)-RACE analyses for the terminal sequences of the RnFV1 genomic RNA and their possible subgenomic RNA(s) were performed as described by Guo et al. (2009) and Suzuki et al. (2004). Nucleotide sequences of selected clones were determined by dideoxy chain termination using an Applied Biosystems 3100 DNA sequencer (Applied Biosystems, Foster City, CA). Single nucleotide positions were read from at least three independent clones. The sequences of deoxyoligonucleotides used as primers are available upon request. DNA sequence data were assembled by Auto Assembler™ DNA Sequence Assembly Software (Applied Biosystems), and analyzed using GENETYX-MAC (GENETYX Co., Tokyo, Japan) and EnzymeX version 3 (http://nucleobytes.com/index.php/enzymex). Full-length RnFV1 cDNA was cloned in pUC57 vector (pUC-N10) that had the T7 promoter sequence immediately upstream of the 5′-terminal end of the RnFV1 genome. The short fragment covering the intergenic and ORF2 region was cloned similarly (pUC-1.7 kb). Sequence analyses of conserved domains was performed using National Center for Biotechnology Information (NCBI) BLAST programs (http://blast.ncbi.nlm.nih.gov/Blast.cgi). Putative transmembrane helices sequences were determined using the TMHMM server version 2.0 (http://www.cbs.dtu.dk/services/TMHMM/) (Krogh et al., 2001). Putative coiled-coil domains were identified with EMBnet COILS (http://www.ch.embnet.org/software/COILS_form.html) (Lupas et al., 1991).

### Database search (*in silico* cloning)

BLAST (tblastn) searches were conducted against sequence databases available from the NCBI (non-human, non-mouse expressed sequence tags, EST; TSA). For the searches, we used RnFV1-NW10 as queries. Plant and fungal transcriptome sequences that matched viral peptides with *E*-values of <0.01 were selected. Obtained sequence fragments were assembled using the Autoassembler software and considerable misreading nucleotides were corrected in a sequence processing program. Reconstructed contigs used in analyses are appended as supplementary data.

### Phylogenetic analysis

Phylogenetic tree construction was based on a maximum-likelihood (ML) method as described previously with minor modifications (Kondo et al., 2013a). The deduced amino acid (aa) sequences of virus and virus-like sequences were aligned with MAFFT version 7 under the default parameters (http://mafft.cbrc.jp/alignment/server/phylogeny.html) (Katoh and Toh, 2008) and gap-removed from the alignment using the program GapStreeze (Los Alamos HIV Sequence Database; http://www.hiv.lanl.gov/content/sequence/GAPSTREEZE/gap.html). The best-fit model for a data set was selected using ProtTest 2.4 server (http://darwin.uvigo.es/software/prottest2_server.html) (Abascal et al., 2005). ML phylogenetic trees were estimated by using PhyML 3.0 under the appropriate substitution mode (http://www.atgc-montpellier.fr/phyml/) (Guindon et al., 2010). The trees were visualized using FigTree version 1.3.1 software (http://tree.bio.ed.ac.uk/software/).

## Results and discussion

### RnFV1 has a single-stranded positive-sense RNA genome

**Figure 1:**
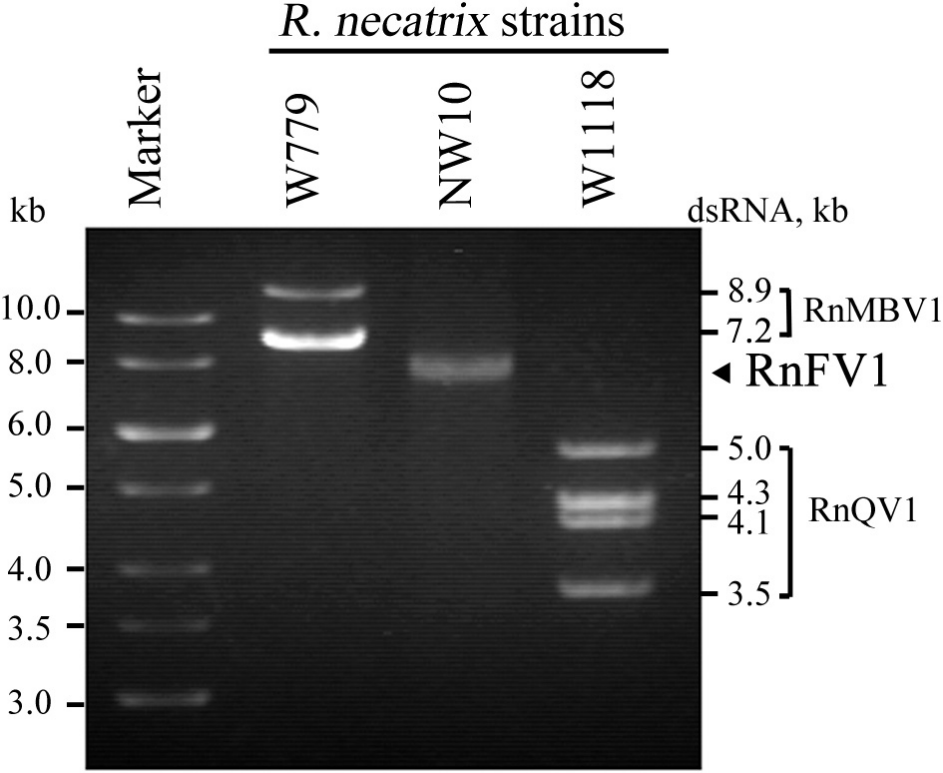
**Gel electrophoretic profiles of dsRNAs isolated from *R. necatrix* NW10**. DsRNAs purified from *R. necatrix* W779 infected with Rosellinia necatrix megabirnavirus 1 (RnMBV1) (lane W779), *R. necatrix* NW10 infected with Rosellinia necatrix fusarivirus 1 (RnFV1), and *R. necatrix* W1118 infected with Rosellinia necatrix quadrivirus 1 (RnQV1) (lane W1118) were analyzed in parallel by 1.2% agarose gel electrophoresis. A GeneRuler 1-kb DNA ladder (Thermo Scientific) was also electrophoresed (Marker).

**Figure 2:**
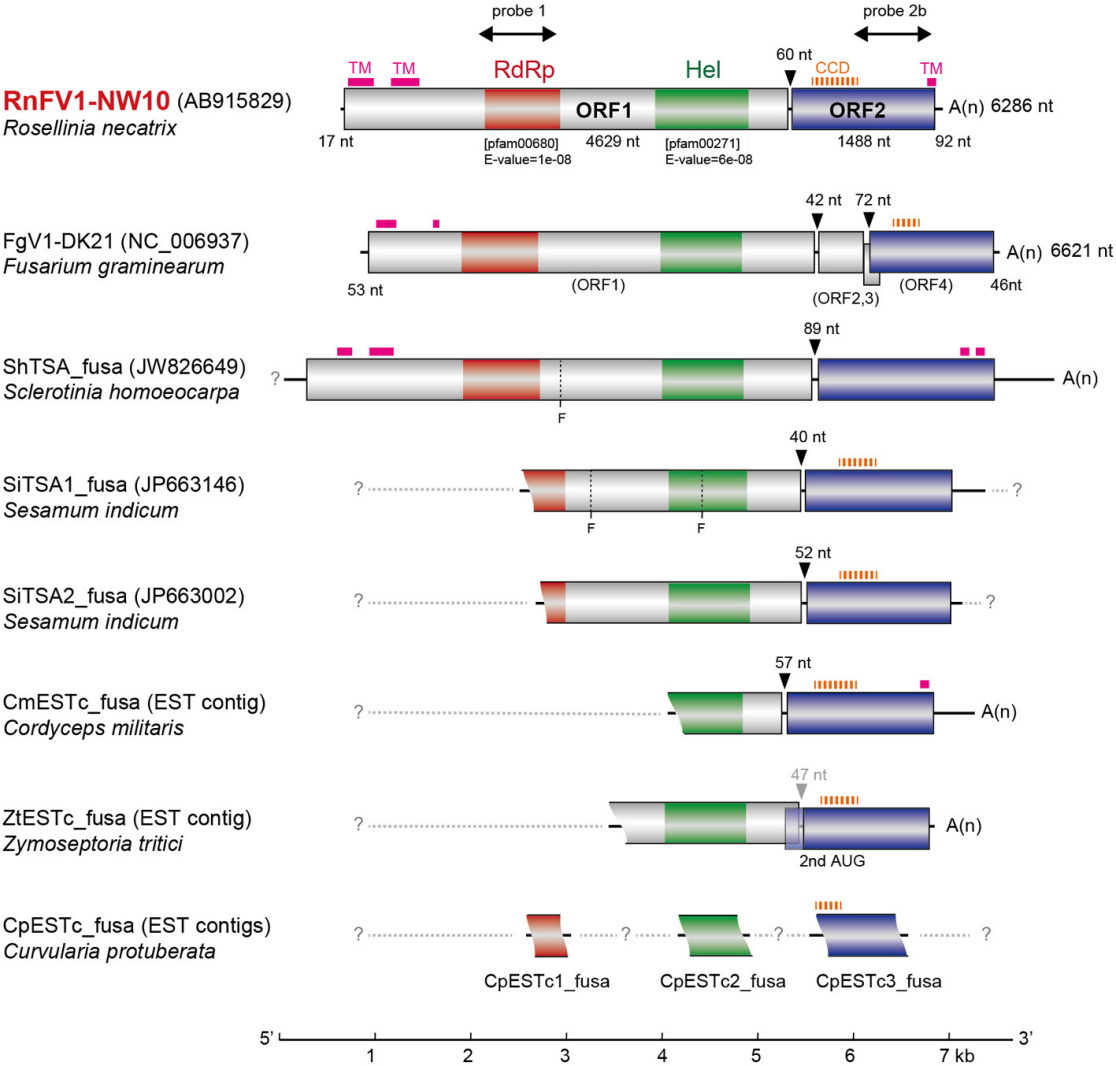
**Genomic organization of RnFV1-NW10 and comparison with those of FgV1 and related virus-like sequences**. The RnFV1 genome is 6286 nts in length excluding the poly(A) tail with two ORFs. The colored boxes and lines represent open reading frames (ORFs) and non-coding sequences, respectively. The boxes in ORF1 represent conserved domains including RNA-dependent RNA polymerase (RdRp, red) and RNA helicase (Hel, green) domains. The sizes of the 5′-UTR, inter-ORF regions, ORF1 and ORF2 are shown above or below the diagram. Regions used for probes in Northern blotting (Figure **4**) are indicated by thick arrows: probe 1, 1440–2180 nt; probe 2b, 5471–6100 nt. The genetic organizations of FgV1 and the assembled contigs of the related virus-like sequences from the transcriptome shotgun assembly (TSA) or expressed sequence tags (ESTs) libraries (see Figure S1 in Supplementary Material) are also similar to RnFV1. Putative transmembrane (TM) and coiled-coil domains (CCD) are denoted by thick pink solid and thick orange dashed lines, respectively (see Figures S2 and S3 in Supplementary Material). The possible undetermined sequences are shown by light-gray dashed lines. Arrowheads indicate the inter-ORF regions and the nt numbers above them denote space. “Fs” at ShTSA_fusa and SiTSA1_fusa denote internal stop codons. A scale bar (1-kb increment) is presented at the bottom.

The RnFV1-NW10 dsRNA purified from fungal strain NW10 showed mobility in agarose gel electrophoresis that corresponded to 6.3 kbp, which was slower than dsRNA1 (5.0 kbp) of RnQV1-W1118, and faster than dsRNA2 (7.2 kbp) of RnMBV1 (Figure 1). The full-length sequence of the dsRNA was obtained by sequencing cDNA clones from a cDNA library generated by a classical non-PCR method and RACE analysis. Two RACE methods (a classical RACE and RLM-RACE) confirmed the terminal sequences 5′-UUUUU—AAAAG-poly(A)-3′. The RnFV1 RNA is 6286 nt long excluding the poly(A)-tail and possesses two ORFs (ORF1 and ORF2) on the genomic RNA (Figure 2). RnFV1 ORF1 encodes a putative 174-kDa protein (1542 aa) with an RdRp (RdRP_1, pfam00680) domain and an RNA helicase (Hel; Helicase_C, pfam00271) domain. These domains were detected using the conserved domain search program on the NCBI web site. The RdRp and Hel domains show moderate levels of sequence identity (66 and 38%, *E*-value = 3e^−109^ and 1e^−41^, respectively) to those of FgV1 and modest levels of identity (28–31%, *E*-value = 1e^−7^–1e^−18^) to the RdRp domain of the prototype hypovirus, Cryphonectria hypovirus 1 strain EP713 (CHV1-EP713), and that of other related viruses (Table 1). Furthermore, these RnFV1 domains also show low levels of aa sequence similarities (<26%) to those of some other (+)ssRNA viruses including plant potyviruses. Interestingly, a low level of sequence identity (approximately 27%) was found between the RnFV1 Hel domain and some bacterial RNA helicases. RnFV1 ORF2 encodes a putative 56-kDa protein (495 aa). Database searches with the ORF2 protein sequence showed no significant similarity to any known viral proteins or recognized protein domains (Table 1). The similarities in the RdRp and Hel regions suggest that RnFV1 is a (+)ssRNA virus.

**Table 1:** **Comparisons of amino acid sequence identity of the motif domains between Rosellinia necatrix fusarivirus 1/NW10 (RnFV1/NW10) and related sequences obtained**.

**Sequence name**	**ORF1**		**ORF2**	**RefSeq/**
	**RdRp (261aa)**	**Hel (278aa)**		**Accession no**.
**VIRUS**				
Fusarium graminearum virus 1/DK21 (FgV1/DK21)	66 (3e^−109^, 93)^a^	38 (1e^−41^, 100)	nh^b^	NC_006937
Cryphonectria hypovirus 3/GH2 (CHV3/GH2)	31 (7e^−17^, 72)	nh	–	NC_000960
Cryphonectria hypovirus 4/SR2 (CHV4/SR2)	30 (3e^−18^, 88)	nh	–	NC_006431
Sclerotinia sclerotiorum hypovirus 1/Sz150 (SsHV1/Sz150)	28 (3e^−15^, 88)	nh	–	NC_015939
Valsa ceratosperma hypovirus 1/MVC8 (VcHV1/MVC8)	30 (1e^−18^, 88)	nh	–	NC_017099
Cryphonectria hypovirus 1/EP713 (CHV1/EP713)	31 (1e^−07^, 44)	nh	–	NC_001492
Cryphonectria hypovirus 2/NB58 (CHV2/ NB58)	31 (3e^−06^, 44)	nh	–	NC_003534
Fusarium graminearum hypovirus 1/NH10 (FgHV/NH10)	28 (3e^−07^, 43)	nh	–	KC330231
Grapevine associated mycovirus-2 (GaMV2)	40 (3e^−64^, 95)	ud^b^	ud/–	GU108600
Grapevine associated mycovirus-3	62 (2e^−55^, 55)	ud	ud/–	GU108601
Barley mild mosaic virus F-SIL (*Bymovirus*; *Potyviridae*)	26 (2e^−05^, 83)	nh	–^b^	AJ544268.1
Chocolate lily virus A (unassigned Secoviridae)	26 (6e^−05^, 75)	nh	–	YP_004936170
**ASSEMBLED VIRUS-LIKE SEQUENCE CONTIG**				
*Sclerotinia homoeocarpa* TSA 13334 (ShTSA_fusa: 8044 nt)	47 (7e^−68^, 99)	38 (1e^−45^, 100)	nh	JW826649
*Sesamum indicum* TSA1 Locus_34152 (SiTSA1_fusa: 4745 nt)	58 (1e^−46^, 60)	38 (6e^−67^, 97)	26 (4e^−24^, 81)	JP663146
*Sesamum indicum* TSA2 Locus_33994 (SiTSA2_fusa: 4289 nt)	52 (9e^−26^, 37)	46 (2e^−55^, 100)	25 (1e^−19^, 83)	JP663002
*Sesamum indicum* TSA3 Locus_33509 (342 nt)	66 (6e^−42^, 37)			JP662560
*Sesamum indicum* TSA4 Locus_34912 (1243 nt)	77 (3e^−18^, 19)			JP663841
*Persicaria minor* TSA PMT0000366898 (475 nt)	66 (4e^−52^, 54)			GALN01314464
*Musa acuminata* TSA AAA group Unigene14017_NK1 (347 nt)	47 (2e^−07^, 27)			JV437227
*Cordyceps militaris* EST contig (CmESTc_fusa: 3407 nt)^c^		45 (6e^−85^, 100)^*^	28 (4e^−46^, 97)^*^	–
*Zymoseptoria tritici* EST contig (ZtESTc_fusa: 3665 nt)^c^		34 (2e^−43^, 86)^*^	25 (2e^−05^, 49)^*^	–
*Curvularia protuberata* EST contig1 (CpESTc1_fusa: 613 nt)^c^	60 (4e^−52^, 52)^*^			–
*Curvularia protuberata* EST contig2 (CpESTc2_fusa: 583 nt)^c^		41 (2e^−45^, 67)^*^		–
*Curvularia protuberata* EST contig3 (CpESTc3_fusa: 1165 nt)^c^			31 (9e^−35^, 52)^*^	–
**BACTERIAL GENE (SELECTED)**				
*Pectobacterium atrosepticum* ATP-dependent RNA helicase HrpB		29 (2e^−09^, 100)		YP_051403
*Vibrio tasmaniensis* DEAD/DEAH box helicase		27 (4e^−09^, 100)		WP_017099829
*Pectobacterium carotovorum* ATP-dependent RNA helicase HrpB		26 (2e^−08^, 100)		YP_006647802

a *The percent of amino acid sequence identity (%) in the sequence comparison analysis is shown with their expect value and query coverage (%) in parenthesis. Each score is calculated by the BLASTp under the default setting*.

* *These scores are calculated by the BLASTp suite-2 sequences with the default setting*.

b *nh, no hit against the corresponding target; ud, undetermined; –, not present*.

c *These contigs are constructed from EST libraries (Figure S1)*.

### RnFV1 is similar in genomic organization to FgV1 and related viral sequences detected by bioinformatic approaches

Database searches of genomes and transcriptomes of cellular organisms have previously revealed novel viral sequences (e.g., Liu et al., 2010, 2012; Chiba et al., 2011; Kondo et al., 2013a). Such approaches allowed detection of as-yet-unraveled negative-strand RNA mycoviruses, and fossil non-retroviral RNA viruses and DNA viruses integrated into fungal genomes. Here, we took a similar bioinformatic approach to detect similar sequences to RnFV1. A search against TSA libraries detected three sequences with a similar genetic organization to RnFV1 in the ascomycete fungus *Sclerotinia homoeocarpa* (ShTSA_fusa, GenBank accession no. JW826649) and sesame plant *Sesamum indicum* (SiTSA1_fusa and SiTSA2_fusa, JP663146 and JP663002) (Table 1). The ShTSA_fusa sequence corresponds well to the entire sequence of RnFV1, while the other two lack the 5′-proximal portions (Figure 2). Some other plant TSA sequences and two short virus-like contigs associated with grapevine plants (Al Rwahnih et al., 2011) also show moderate levels of aa sequence identity with RnFV1 RdRp domain (Table 1). Using the RnFV1 sequence as a query, a BLAST search against expressed sequence tag (EST) libraries was also able to detect a similar Hel domain and ORF2 sequences in several fungal strains that correspond to the 3′-proximal region or discontinuous multiple portions. Examples include: CmESTc_fusa from *Cordyceps militaris*, an important ascomycetous medicinal mushroom (caterpillar fungus); ZtESTc_fusa from *Zymoseptoria tritici*, an ascomycete causing septoria tritici blotch in wheat; CproESTc1_fusa–CproESTc3_fusa from *Curvularia protuberata*, an ascomycetous host of Curvularia thermal tolerance virus conferring heat tolerance to tritrophic interaction partners (panic grass and the fungal host) (Márquez et al., 2007) (Figure 2) (Table 1).

These identified sequences are likely of viral origin, and not of host origin for several reasons. No such sequences can be detected in available genome databases for the fungi and plants (data not shown). Furthermore, sequence comparison among (+)ssRNA mycoviruses and the data-mined, assembled virus-like contigs (Figures S2 in Supplementary Material) showed interesting common properties. Higher interviral sequence identity was found between RnFV1 and CmESTc_fusa or SiTSA_fusa than any other pair-wise comparisons, as summarized in Table 1. All of these related sequences appear to have a similar genetic organization, harboring two potential ORFs, except for the internal stop codon at ShTSA_fusa and SiTSA1_fusa (denoted by F in Figure 2), which encoded the putative replicase and unknown proteins. The only exception is FgV1, which has four ORFs; the two small ORFs (ORFs 2 and 3) of FgV1 are observed only in FgV1, but no ORF larger than 250 nt is detected in the other assembled virus-like sequences in their corresponding positions (Figure 2). The RdRp and Hel domains are found in a relatively similar location and order in the ORF1 of ShTSA_fusa, SiTSA_fusa and two mycoviruses (RnFV1 and FgV1). Transmembrane (TM) domains (Figure S2 in Supplementary Material) are also found at the N-terminal portion of ORF1-coded protein of RnFV1, FgV1, and ShTSA_fusa using a bioinformatic tool (TMHMM) (Krogh et al., 2001). The ORF2 proteins of the virus-like sequences show modest levels of sequence similarity (25–31% identity) to that of RnFV1 (Table 1). It should be noted that these ORF2 proteins showed similar structure profiles predicting a coiled-coil domain (CCD) (all proteins except for ShTSA_fusa) and a TM domain (RnFV1, ShTSA_fusa, and CmESTc_fusa) at the N-terminal to central regions and the C-terminal regions (Figure 2), respectively, when predicted by bioinformatic tools COILS (Lupas et al., 1991) and TMHMM (Figure S3 in Supplementary Material and data not shown). Although viruses as a physical entity have yet to be identified, these data support the above hypothesis and possible prevalence of similar viruses in diverse host organisms from fungi to invertebrates. Alternatively, the related sequences may be from fungi associated with host plants and invertebrates.

### Phylogenetic analysis of RnFV1 and their relatives

**Figure 3:**
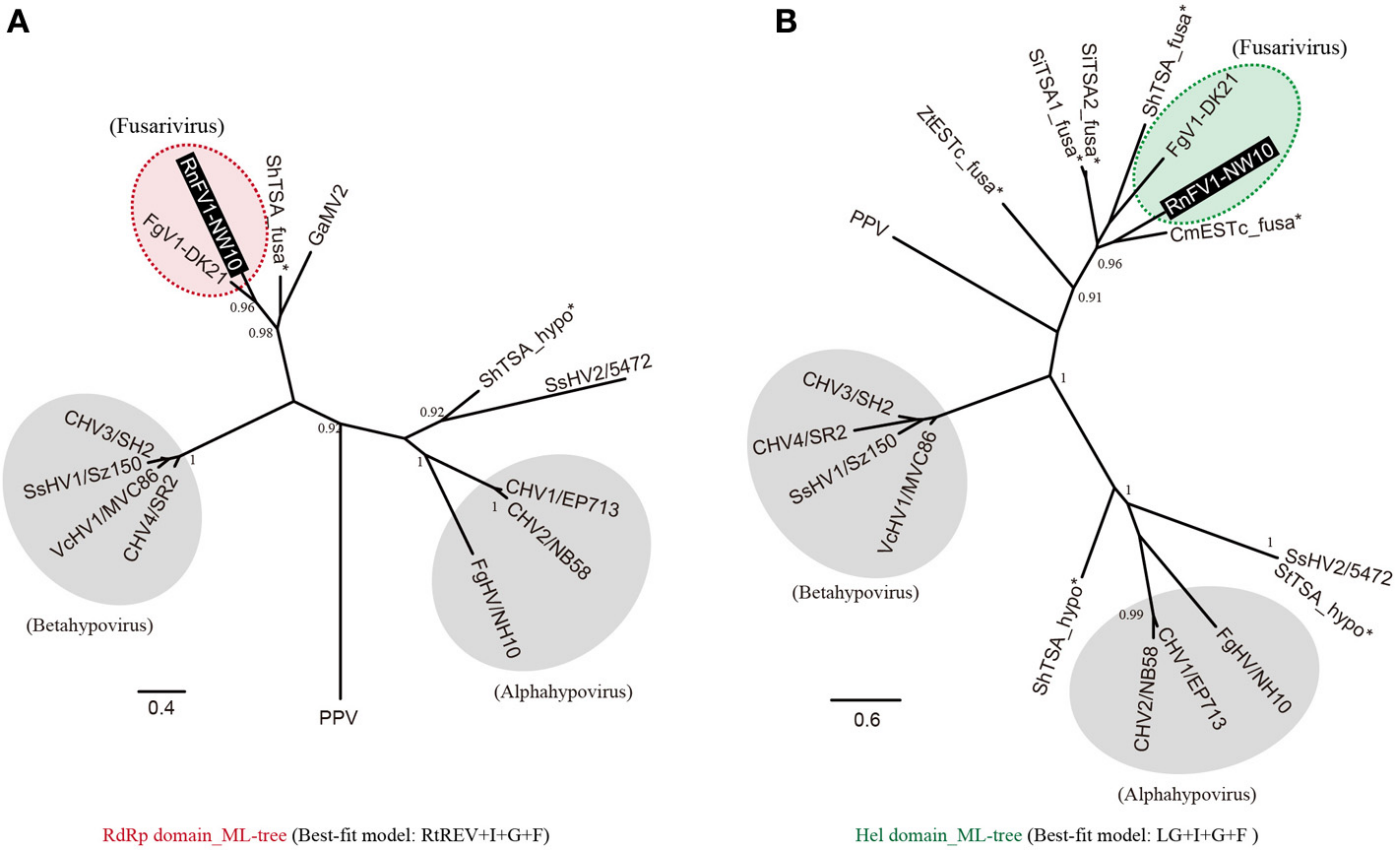
**Maximum likelihood (ML) phylogeny of RnFV1-NW10 and its related virus and virus-like sequences**. Phylogenetic trees were constructed using PhyML 3.0 based on the multiple aa sequence alignment of the RNA-dependent RNA polymerase (RdRp) **(A)** and RNA helicase (Hel) domains **(B)**, as shown in Figure S4A and B, respectively, in Supplementary Material. The cured alignments were subjected to phylogenetic analysis. The selected best-fit models for each alignment are shown at the bottom. Red and green shaded circles with dashed line: fusariviruses proposed in this study. Gray circles: the members of the genera *Alphahypovirus* and *Betahypovirus* proposed by Yaegashi et al. (2012). The related virus-like sequences denoted by asterisks were also included in the analyses. GenBank/Refseq accession numbers of the sequences are listed in Table 1 with the addition of PPV (plum pox virus-NAT; D13751), Sclerotinia sclerotiorum hypovirus 2 isolate 5472 (SsHV2; NC_022896), ShTSA_hypo (*Sclerotinia homoeocarpa* TSA sequence; JW820052) and StTSA_hypo (*S. trifoliorum* TSA sequence; JP556778). Asterisks show the virus-like sequences. The branch support values were estimated by the approximate likelihood ratio test (aLRT) with a SH-like algorithm (Anisimova and Gascuel, 2006) (only values greater than 0.9 are shown).

As shown above, sequence similarities were detected between RnFV1 and other (+)ssRNA mycoviruses including hypoviruses and FgV1. In order to assess the relationships among them, we conducted a phylogenetic analysis with the assembled virus-like sequences (Figure 2), based on aa alignments of well-conserved domains (RdRp and Hel) of these virus and virus-like sequences. The phylogenetic tree generated from the RdRp alignment shows that RnFV1 clusters together with FgV1 and ShTSA_fusa distinctly from hypoviruses and plum pox virus (PPV, a plant potyvirus) used as an outgroup (Figure 3A). A similar tree topology was observed in a Hel-based ML-tree (Figure 3B).

### Attempts to purify virus particles

Most fungal RNA viruses can be classified into two groups: real dsRNA viruses and pseudo-dsRNA viruses (Hillman and Suzuki, 2004). Viruses of the second group are now regarded as (+)ssRNA viruses that include the families *Hypoviridae, Narnaviridae*, and *Endornaviridae* (Ghabrial and Suzuki, 2009). Members of this group commonly lack the ability to form rigid virus particles but instead induce Golgi-derived lipid-vesicles. Here, we tested whether RnFV1 is able to form particles. However, we failed to purify RnFV1 virions by conventional methods which allowed for isolation of megabirnaviruses and quadriviruses known to be able to form virions. The most closely related virus, FgV1, was reported to not form rigid virus particles by Kwon et al. (2007). Our failure to purify particles may suggest a common property shared by these related mycoviruses.

### Attempts to detect RnFV1 ORF2 subgenomic RNA

**Figure 4:**
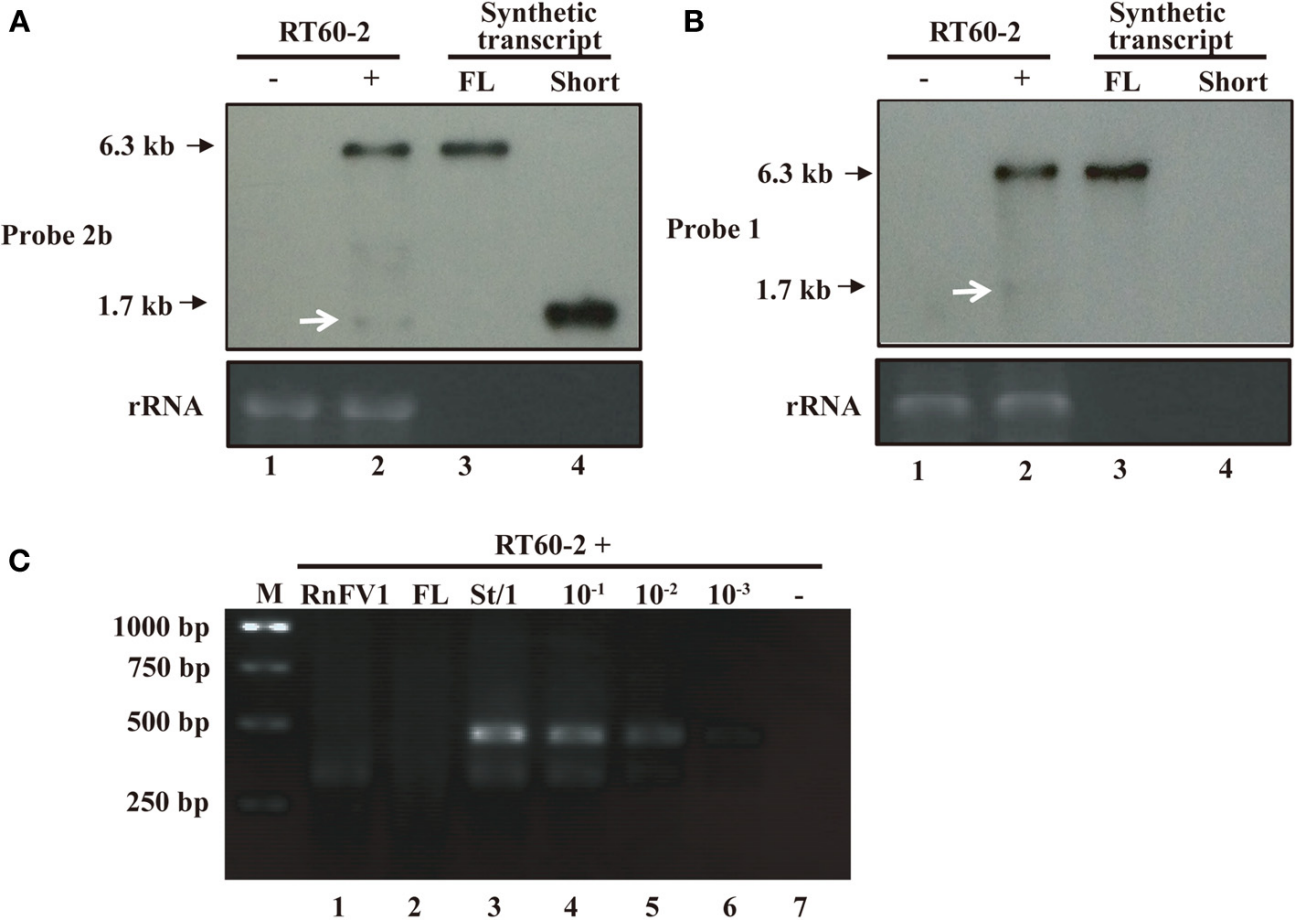
**Northern blotting (A,B) and 5′ RACE analyses (C) of RT60-2/RnFV1**. Total RNA fractions (0.5 μg/lane), obtained from virus-infected RT60-2/RnFV1 (+) (lane 2) and virus-free RT60-2 (−) (lane 1), were electrophoresed under denaturing conditions and subjected to Northern blotting as described in Materials and Methods **(A,B)**. *In vitro* synthesized, 6.3-kb genome-sized (lane 3, FL, 10 ng) and 1.7-kb shorter-sized transcripts (lane 4, Short, 0.1 ng) were analyzed in parallel **(A,B)**. The short transcript corresponds to positive-sense RNA spanning ORF2 plus the 3′-UTR region (see Figure 5A for *in vitro* transcription start site G at position 4652) and mimics hypothetical subgenomic RNA. Two cDNA probes specific for regions of ORF1 (probe 1) and ORF2 (probes 2b) (see Figure 2 for positions) were used in B and A, respectively. Faint bands detected at a similar position to the short transcript by each probe (shown by white arrows), these are unlikely to be subgenomic RNA (see the text and Figure 4C). Classical 5′-RACE, entailing primer-specific cDNA synthesis, dC-tailing, and PCR amplification, was conducted aiming at subgenomic RNA detection **(C)**. The template was 0.5 μg of total RNA from virus-infected RT60-2 (lane 1). As controls, 0.5 μg of total RNA from virus-free RT60-2 without (−, lane 7) or with ten-fold serial dilutions of short (St) 1.7 kb RNA, ranging from 2.6 ng to 2.6 pg (lanes 3–6), was analyzed in parallel. Two viral specific primers 5′-CTTGTATTGCTTGCGGGAGGCTTCC-3′ (map positions 5102-5078) and 5′-TCAAGCGAAGAGAGTTCAATTC-3′ (map positions 4989-4968) were used for cDNA synthesis and DNA amplification. Similarly 10 ng of genome-sized transcript (lane FL) was used in this assay. PCR products were examined by 1.5% agarose gel electrophoresis.

It is important to determine how the downstream ORF of RnFV1 is expressed. It was reported that FgV1, evolutionarily related to RnFV1, had three ORFs in the 3′-proximal portion of the genome that are expressed via subgenomic RNAs (Kwon et al., 2007; Cho et al., 2013). Based on the sequence similarity, we anticipated that RnFV1 uses the same expression strategy for ORF2. In order to examine this possibility, we first performed a Northern analysis with a digoxigenin (DIG)-11-dUTP-labeled DNA probes. In this assay known amounts of *in vitro* synthesized transcripts of a genome size (6.3 kb, FL-transcript) and a size expected for possible subgenomic RNA, 1.7 kb (short-transcript), were used in parallel as reported by Guo et al. (2009). As a result, a major RNA band with the same mobility as FL-transcript was detected specifically in RnFV1-infected RT60-2 (RT60-2/RnFV1, see below), but not in virus-free RT60-2, using an ORF2-specific probe (Figure 4A). In addition, a faint RNA band was detected in RnFV1-infected fungal strains (Figure 4A, lane 2, shown by white arrows) at a slightly faster migration position relative to the *in vitro* synthesized RNA (Figure 4A, lane 4). An ORF1-specific probe also detected a faint RNA band below the 18S rRNA (Figure 4B, lane 2, shown by white arrows) in addition to the major genome-sized RNA. Because such RNA bands often appear unexpectedly in other systems (Chiba et al., 2013b), these RNAs were considered not to be subgenomic RNA of RnFV1.

Next, we carried out classical 5′-RACE taking the same approach as for CHV1 (Guo et al., 2009) in which a lack of subgenomic mRNA for its downstream ORF B was confirmed. As expected from the above Northern analysis, no RACE products were obtained (Figure 4C, lane 1) under conditions where 2.6 pg of *in vitro* synthesized RNA gave amplified fragments. Note that 10 ng of genome-sized synthetic transcripts in 0.5 μg of total RNA represents similar accumulation levels of RnFV1 full-length transcript in infected mycelia (Figures 4A,B). This amount (2.6 pg) is approximately a 1/1000 molar ratio of viral genome-sized (+)RNA present (Figure 4A) in 0.5 μg of the total RNA fraction. Sequence analyses of cloned RACE products from the synthetic transcripts (Figure 4C, lane 3) showed that the amplified major longer and minor shorter DNA fragments corresponded to fragments that entirely or partially covered the 5′-terminus of the 1.7-kb synthetic RNA. Overall, it is suggested that ORF2 subgenomic RNA is unlikely to be produced in infected mycelia, but we cannot rule out the possibility of subgenomic RNA production below the detection level of the RACE used here.

### Sequence similarity in intergenic regions among RnFV1, related viruses and virus-like contigs

**Figure 5:**
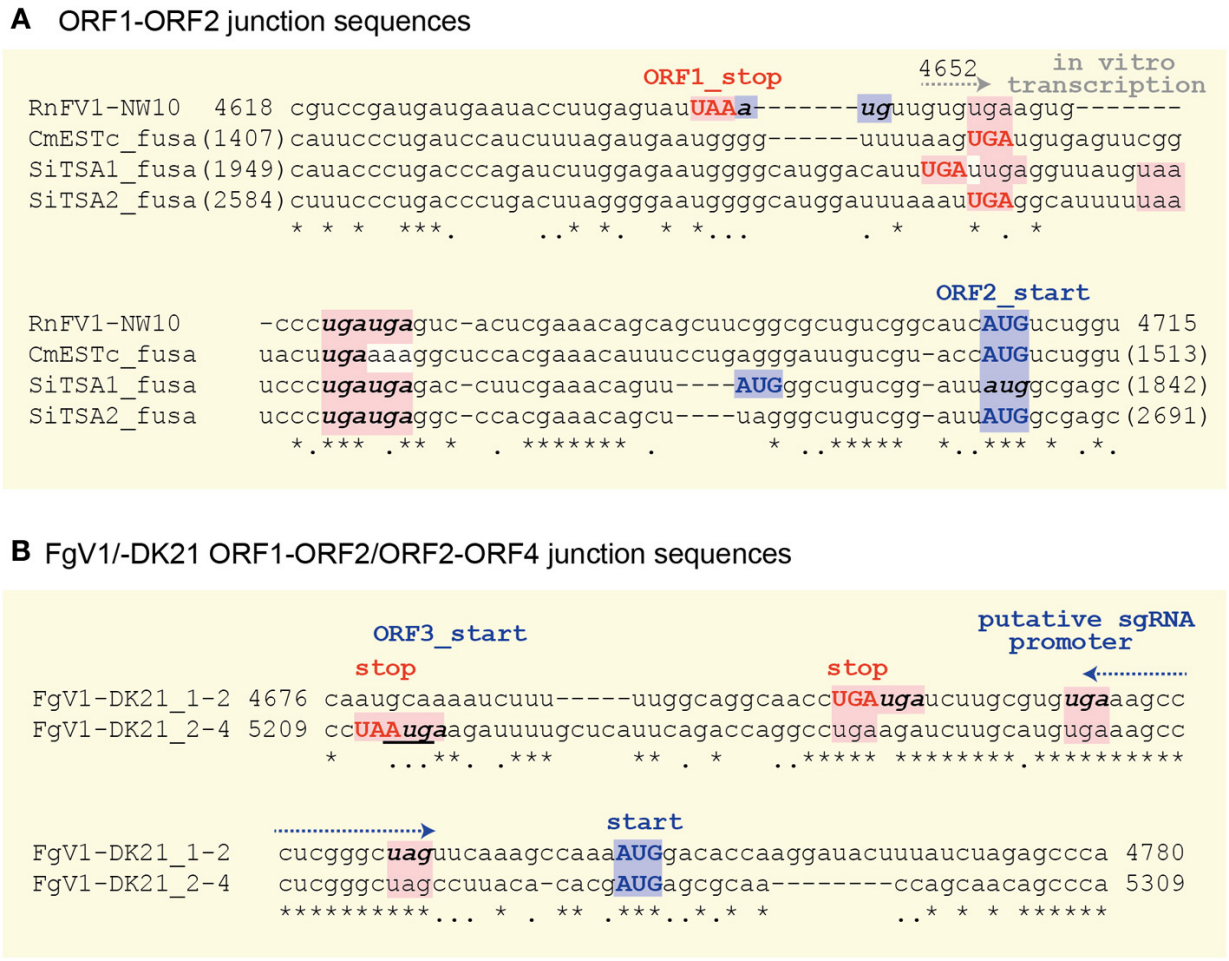
**Comparison of the inter-ORF sequences and their flanking regions between RnFV1-NW10 and the selected virus-like sequences (A) or within FgV1 (B)**. The upper-case bold letters in red and blue indicate the stop codon of the upstream ORF and the start codon of the downstream ORF, respectively. The in-frame stop or start codons in the inter-ORF regions are denoted by bold, lower-case italic letters except for the in-frame downstream AUG codon of SiTSA1_fusa. The ORF3 start-codon that overlaps the ORF2 stop codon in FgV1 is underlined. Asterisks, dots, and — indicate identical and similar nucleotides, and gaps, respectively. All potential start and stop codons are highlighted by pink and light blue, respectively. The G at position 4652 for *in vitro* transcription start site is indicated (see Figures 4A,B).

There are highly conserved sequence stretches in intergenic regions among all related viral sequences except for FgV1. A closer look at the intergenic sequences of RnFV1 and related viral sequences showed similarity among them (Figure 5). There are at least two stop codons downstream of ORF1, eliminating the possibility of a read-through translation of the downstream ORFs that are situated either in-frame, in −1 frame or in −2 with ORF1. Interestingly, FgV1 has its own conserved intergenic sequence distinct from the above viral/database-mined sequences that may serve as a regulatory sequence for subgenomic RNA synthesis (Kwon et al., 2007). An interesting similarity extends to the intergenic length (40–89 nt) of the two ORFs in these sequences. Note that two ORFs of ZtESTc_fusa partially overlap if its first AUG is used as a start codon. Of particular note is that while 5–7 nt stretches with 100% identity were found among the inter-ORF sequences in RnFV1, CmESTc_fusa, SiTSA1_fusa, and SiTSA2_fusa (Figure 5A), a longer stretch with high sequence identity was detected between the two inter-ORF sequences of FgV1 (ORF1/ORF2 and ORF2/ORF4) (Figure 5B). These conserved sequence features lead us to speculate that they serve as promoters or enhancers for as-yet-unidentified subgenomic RNAs or non-canonical translation from a genome-sized mRNA.

### Biological comparison of Rnfv1-infected and virus-free strains of *R. necatrix*

**Figure 6:**
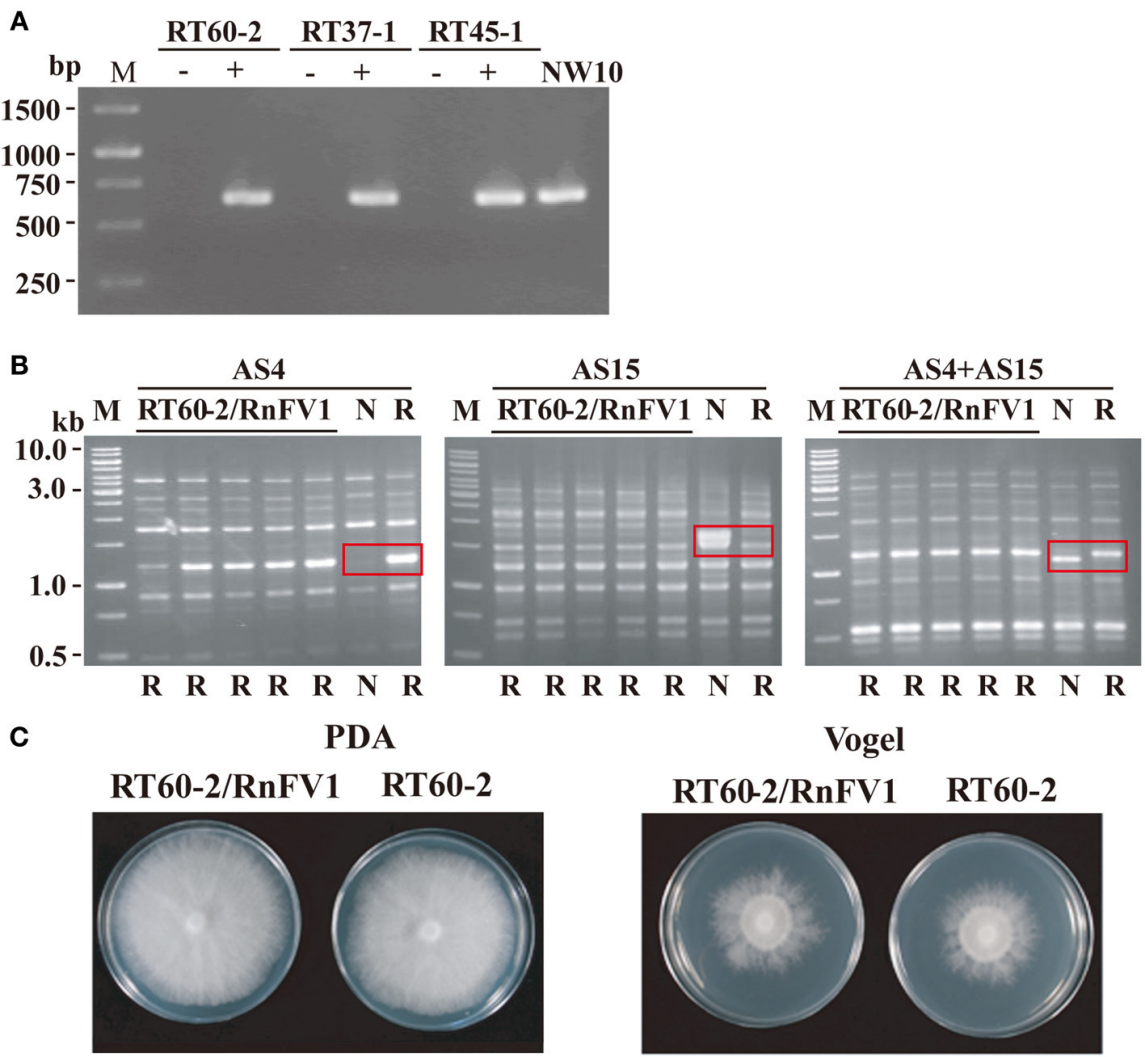
**Colony morphology of RnFV1-infected and virus-free isogenic strains**. RnFV1-NW10 was transferred from *R. necatrix* NW10 to somatically incompatible fungal strains (RT60-2, RT37-1, and RT45-1) via co-culturing in the presence of zinc chloride. Infection of the fungal recipient strains by RnFV1 was confirmed by RT-PCR **(A)**. Total RNA fractions, isolated from RT60-2, RT37-1, and RT45-1 before (−) and after (+) virus transmission, were used in RT-PCR. The donor strain NW10 was also included in the analysis. An oligo (dT) was used as a primer for cDNA synthesis, while a forward primer 5′-CATTCGCAAGAGCCTGAGC-3′ (map positions 5471-5489) and a reverse primer 5′-AGCCAACGCCTAACAAGCAC-3′ (map positions 6100-6081), were used as primers for DNA amplification. The genetic backgrounds of recipient fungal strains were examined by UP-PCR using three primer sets (AS4, AS15, and AS4+AS15) **(B)**. All recipient isolates (RT60-2/RnFV1) manifested UP-PCR profiles identical to their original strain, RT60-2 (lane R), and not to NW10 (lane N), confirming lateral virus movement. Abbreviations (N and R) below each gel indicate the banding pattern for their parental strains. Distinguishing banding patterns of two parental strains are boxed in red in each UP-PCR gel. A GeneRuler 1 kb DNA ladder (Thermo Scientific, M) was also electrophoresed. As an example, virus-uninfected (RT60-2) and virus-infected (RT60-2/RnFV1) fungal strains were grown on PDA and Vogel's medium for 6 days in the dark and photographed **(C)**.

We first attempted to eliminate virus from *R. necatrix* strain NW10 via hyphal tipping. Nevertheless, repeated attempts were unsuccessful, similar to the case for eliminating a victorivirus in *R. necatrix* strain W1029 (Chiba et al., 2013a) and unidentified viruses in *R. necatrix* strain W1032 (data not shown). Thus, we took a different approach to determine a cause/effect relationship, i.e., a zinc ion-mediated method for delaying programmed cell death as an incompatible mycelial response (Ikeda et al., 2013). Three hygromycin-resistant *R. necatrix* strains (RT60-2, RT45-1, and RT37-1), which are mycelially incompatible with each other and with NW10, were used as recipients. Several subcultures were obtained from the recipient side of each pair and cultured on PDA plate with hygromycin (PDA-Hyg) repeatedly. Horizontal virus transfer to the recipients was confirmed by RT-PCR (Figure 6A), while their genetic backgrounds were shown by UP (universally primed)-PCR to be the same as the recipient strains (Figure 6B and data not shown). Namely, resultant fungal strains showed the same UP-PCR patterns as those of the recipient parent strains, which were dissimilar to those of the donor strains. Representative UP-PCR results of subcultures obtained from a pairing between NW10 and RT60-2 are shown in Figure 6B. Recipient subcultures (RT60-2/RnFV1) had the same UP-PCR profiles as those of the original RT60-2 but not of NW10 (Figure 6B). This resulted in three sets of virus-free and virus-infected fungal strains: RT60-2 and RT60-2/RnFV1, RT45-1 and RT45-1/RnFV1, and RT37-1 and RT37-1/RnFV1. A comparison of colony morphology between virus free and virus-infected isogenic strains grown nutrition-rich (PDA) and -poor (Vogel's) media suggest asymptomatic infections (Figure 6C and data not shown). This notion was supported by a virulence assay that showed no significant difference between these fungal strains on apple rootstocks (data not shown).

This zinc ion-mediated method was previously used for lateral transfer of several mycoviruses in *R. necatrix* such as RnMBV1 and a partitivirus (Ikeda et al., 2013). Here we provide an additional example, RnFV1 suggesting its wide applicability as long as the white root rot fungus is used as the host.

## Conclusion

Approximately 20% of collected field isolates of *R. necatrix* contains different patterns of dsRNAs considered to be viral genomes (Arakawa et al., 2002; Kondo et al., 2013b). All *R. necatrix* viruses characterized previously have dsRNA genomes, and are highly likely to form rigid virus particles (Kondo et al., 2013b). RnFV1 is the first (+)ssRNA virus of *R. necatrix* whose genome has been thoroughly characterized. Similar to some mycoviruses such as hypoviruses, narnaviruses, and endornaviruses, RnFV1 is most likely unable to form virus particles because no fraction after sucrose gradient centrifugation, which is commonly used for virion purification, contained particles.

We failed to isolate virus-free strains via hyphal tipping which previously allowed us to eliminate quadriviruses and partitiviruses (Lin et al., 2012, 2013; Chiba et al., 2013a), suggesting that RnFV1 is maintained stably and systemically in this natural fungal host. This notion is strengthened by an interesting phenomenon previously reported by Yaegashi et al. (2013). The fungal strain NW10 was introduced into the soil of an experimental orchard of the Nagano Fruit Tree Experiment Station (Suzaka, Nagano, Japan) as a bait or source to examine whether it provided or received this or any other mycovirus extracellularly. All sub-isolates of NW10 collected from the plot 3 years after treatment, carried RnFV1. In addition, RnFV1 was transmitted to mycelially incompatible fungal strains (e.g., an originally virus-free strain W563, MCG139) in the soil. This fact indicates that the virus is stably retained and spread in the soil with the host fungus under natural conditions.

RnFV1 is most closely related to FgV1, which has not yet been assigned to any genus or family by the ICTV. As noted before (Kwon et al., 2007; Cho et al., 2013), these viruses are similar to members of the family *Hypoviridae*. Similarities to hypoviruses are found in the aa sequences of RdRp and Hel domains, their order, existence of poly(A) tails and possible inability to form rigid particles. Dissimilarities include the length of the 5′-UTR (193–487 nt for hypoviruses vs. 18–53 nt for FgV1 and RnFV1), position of small ORF(s) and their aa sequences (e.g., lack of a papain-like protease domain in the FgV1 and RnFV1 ORF1 proteins, which is commonly found in hypoviral polyproteins), and genome size (9.2–12.7 kb for hypoviruses vs. 6.3–6.6 kb for FgV1 and RnFV1) (Hillman and Suzuki, 2004; Cho et al., 2013; Wang et al., 2013). Similarities between RnFV1 and FgV1 include high levels of sequence identities found in the RdRp and HEL domains of ORF1, the poly(A) tail, the gene arrangement, and the genome size (6.3 vs. 6.6 kb). Such commonalities and their close phylogenetic relationship lead us to propose the creation of a novel family designated *Fusariviridae* named after the original host *Fusarium graminearum* for FgV1. Although the proposed family currently accommodates only two members, ICTV-approved *Fusarium graminearum virus 1* and a novel mycovirus *Rosellinia necatrix fusarivirus 1* (this study), its members are expected to increase from the BLAST search results shown in Table 1. An interesting difference is the number of ORFs: four for FgV1 vs. two for RnFV1. Another noteworthy difference is their ability to affect host fungi; while FgV1 reduces production of the mycotoxin and virulence of *F. graminearum* (Cho et al., 2013), RnFV1 infects the natural host asymptomatically. Recently, the Woronin body (a dense-core vesicle specific to filamentous ascomycetes) component, HEX1, required for the virulence and asexual sporulation of *F. graminearum*, was shown to enhance FgV1 replication (Son et al., 2013). It will be of interest to investigate whether the *hex1* gene ortholog of *R. necatrix* also plays a role in RnFV1 replication.

### Conflict of interest statement

The authors declare that the research was conducted in the absence of any commercial or financial relationships that could be construed as a potential conflict of interest.
