# Supplementary material for: A novel single-stranded RNA virus isolated from a phytopathogenic filamentous fungus, Rosellinia necatrix, with similarity to hypo-like viruses
Source: Front Microbiol. 2014 Jul 18;5:360. doi: 10.3389/fmicb.2014.00360 (PMC4103508; doi:10.3389/fmicb.2014.00360)
Supplement: Supplementary file 1 [file DataSheet1.PDF]

Cordyceps militaris EST contig (CmESTc_fusa: 3407 nt)

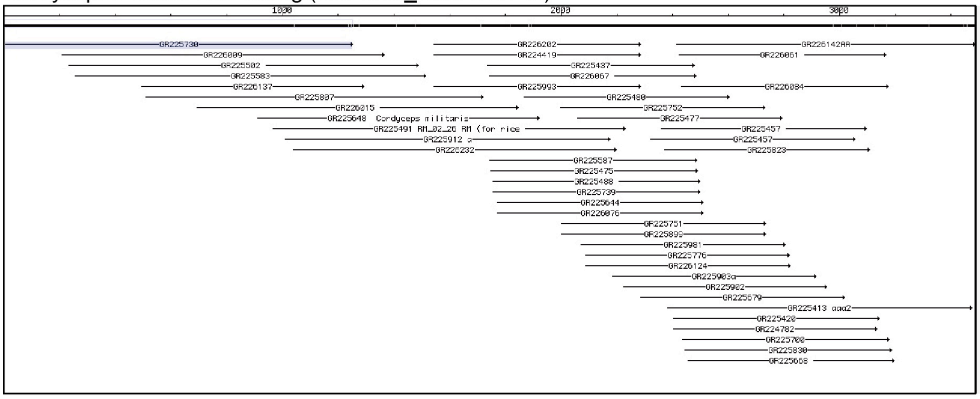

Zymoseptoria tritici EST contig (ZtESTc_fusa: 3667nt)

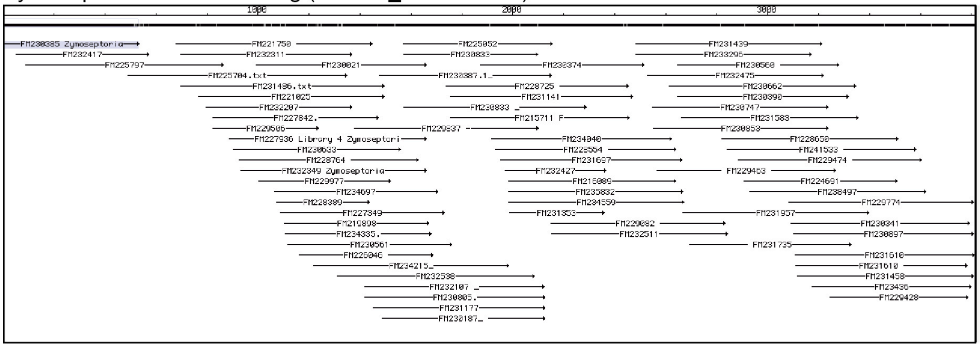

Curvularia protuberata EST contig1 (CpESTc1_fusa: 613 nt)

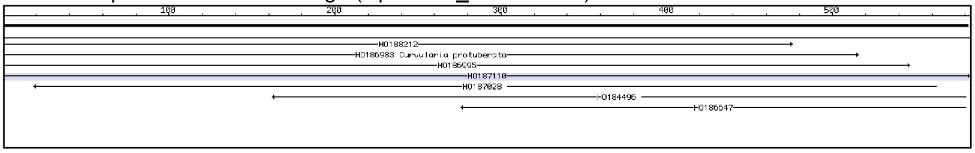

Curvularia protuberata EST contig2 (CpESTc2_fusa: 583 nt)

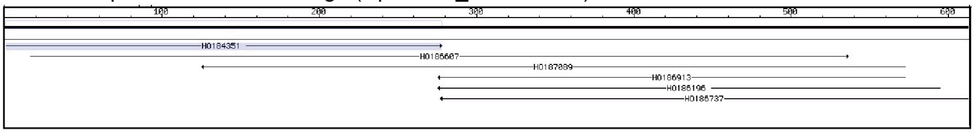

Curvularia protuberata EST contig3 (CpESTc3_fusa: 1165 nt)

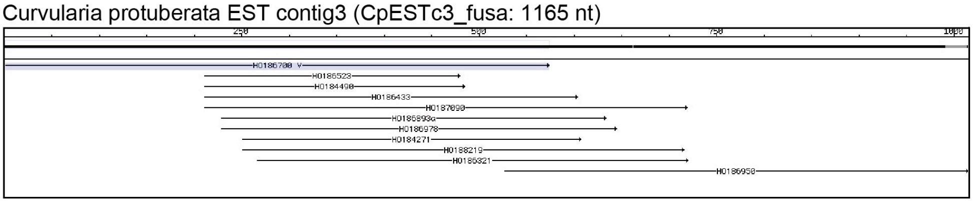

Figure S1. Virus-like sequences assembled from EST libraries of different fungi. RnFV1/NW10-related sequences were detected by searching EST databases for *Cordyceps militaris*, *Zymoseptoria tritici*, and *Curvularia protuberata*. Multiple ESTs were used to construct contigs where overlapping regions of EST sequences show approximately over 99% sequence identity.

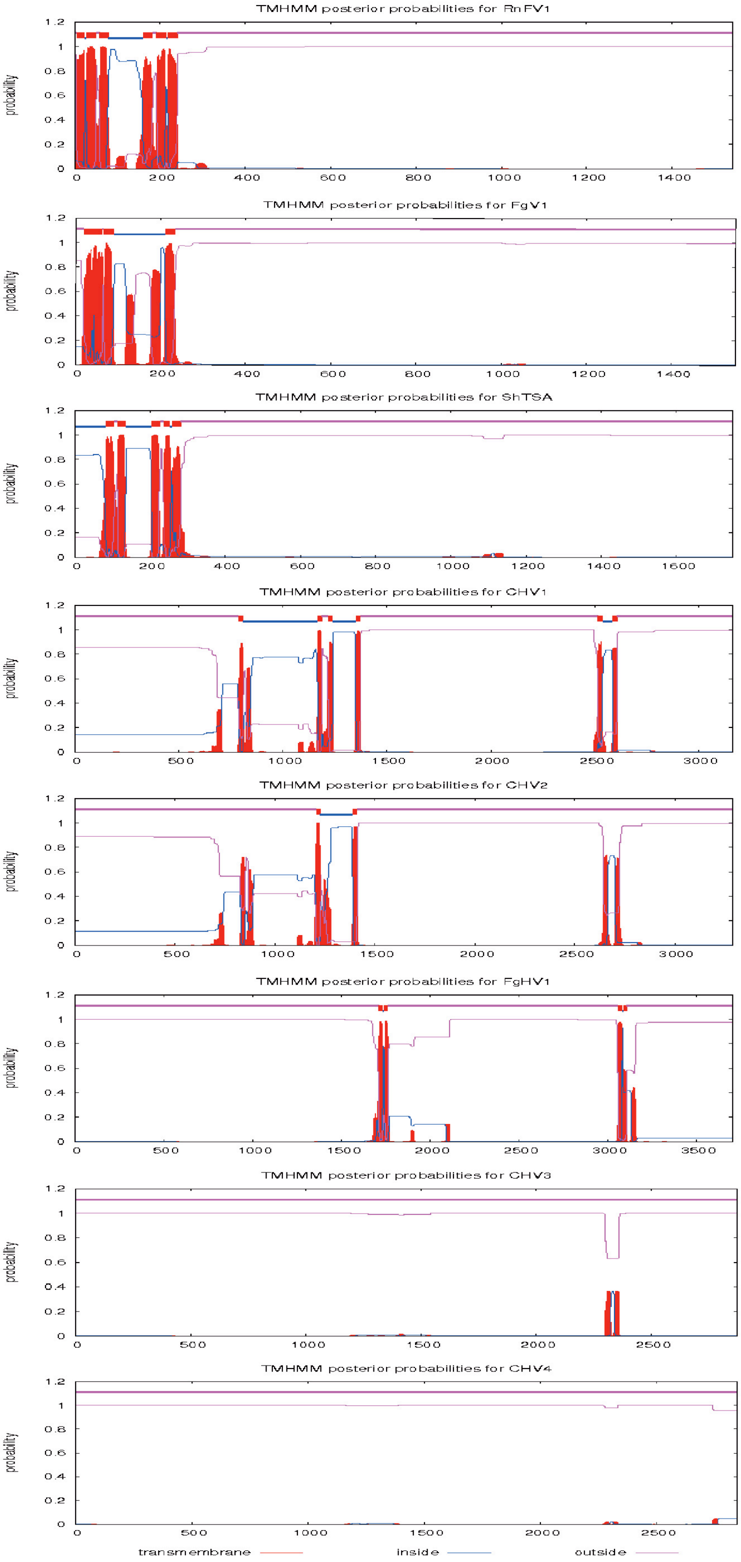

Figure S2. Transmembrane domains prediction for ORF1 of RnFV1/NW10 and related viruses and a virus-like sequence.

A

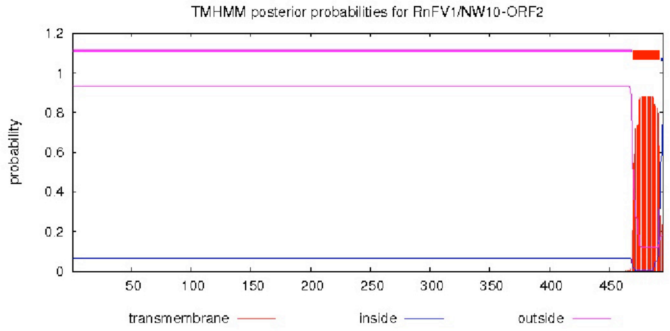

B

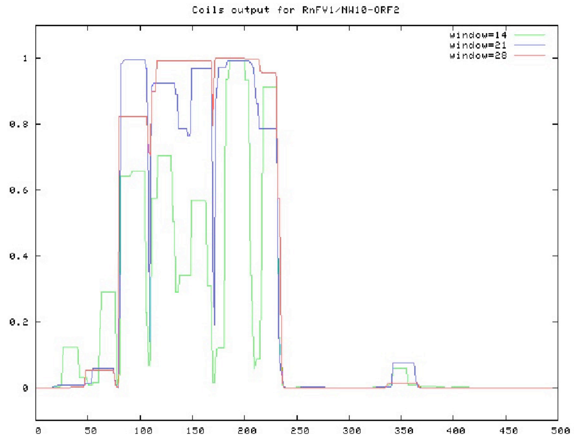

Figure S3. Transmembrane (A) and coiled-coil (B) domains prediction for RnFV1/NW10 ORF2 protein.

A: RdRp domain

```
CLUSTAL format alignment by MAFFT (v7.146b)


FgHV/HN10B      FVKSQITNAVKAMPKSVGGQDKQFRTVVAQDDFSYMQNQVVMMDRNKRDWADEF--GAGA
CHV1/EP713      FAKSQAVPGQPLLAPRM----KDLRTVVSEDLSAYMVDQIFQIEANKRITWETY--GAGS
CHV2/NB58       FAKSQAVDGTALLPPKL----KDLRTVVSQDISSYFVDQIFQIEANKRITWETY--GAGS
ShTSA_hypo      FIKSQVVDIGKVLDKA-----KNVRTVVAEDLVTYFMNQAIELERNKRHVPDA---GFGM
SsHV2_5472      KVKGQAVPVAPLVTPNADGLYKPVRTFVAQDSRSTAIDWTVGLELKNRLPGDHV--GITS
CHV3/GH2        FIKDEVLPKSYVER-------EKIRTIIAQDPLNYYLSMAVQGDAAKRLDPSSF---SAV
SsHV1/Sz150     FIKDEVLPASYIER-------EKIRTIIAQDPLNYFLAMAVQGDGAKRLDPSSF---SAV
VcHV1/MVC86     FIKDEVLPMSYVER-------EKIRTIIAQDPLNYYLSMAVQGDAAKRLDPNSF---SAV
CHV4/SR2        FIKDEVLPESYIER-------EKIRTIIAQDPLNYYLSMAVQGDQAKRLDPLSF---SAV
FgFV/DK21       SVKGEALPPKKWME-------DKVRTIIGTPLVHYIMTTIWNYEPNHRFAWVST--PTKI
RnFV1-NW10      SVKDEALPAKKWME-------DKLRTVIGSPLGQYIMSTIWNYAPNHNFKFDST--PIKV
ShTSA_fusa1     FYKSEALPEKKWRN-------NKVRTPIGSMLPQYLWQMVWSYEPNHRFDPENT--PIKI
GaMV2           FTKAEFLPPRKWQK-------GKVRTPVASMLPQYISQMVWSGVQNHRFRPFDT--PVKI
PPV             SLKAELRPKEKVEA-------NKTRSFTAAPIDTLLGGKVCVDDFNNQFYSLNLHCPWSV
                  * :                   *:  .                 :.

FgHV/HN10B      GMRLNQ-SMLKNFLALEEPKETFEGLYMMADATAFDSTIPNIIQCYHERLWSHGFKNHPS
CHV1/EP713      GMPLSQ-SMARIWDELHDLRKREGGQFIIADATAYDSNCKPALFHGAGKLVELGFQNHPS
CHV2/NB58       GMPLSQ-AMARIWDELHDLRLREGGQFIIADAKAYDSKCKPALFLGAGRLVERRFANHPS
ShTSA_hypo      GMVLNQ-NMAALFKALADHCDATGGVLTFADATEFDSKTAPSMWRMLSRLGELGLKDQV-
SsHV2_5472      KMAAGQ-GYSNLFRRIR-----GKENIFMGDMGQYDSRLEREHFYMLDRALENGVSDKI-
CHV3/GH2        GVSASHGEMSALAEKHL-----AYKHHTAMDVTAMDSTASIDAVGVIKKLRKKGFQKHS-
SsHV1/Sz150     GISPSHGQMSALAAKHL-----AYEHHTAMDVTAMDSTAAIDAVGVIKKLNKKGYKNHP-
VcHV1/MVC86     GVSASHGEMSALAEKHL-----AYKHHFAMDVTAMDSTASVDAMGVIKKLRKKGFQDHP-
CHV4/SR2        GVSPAHGEMSALAERHL-----AYKHHFAMDITAMDSTAAVDCVGVIKRLRKLGFKHHP-
FgFV/DK21       GMPLNGYWLSDLYFRHS-----RCQHHFAGDMSSFDSTLSGKVIELVKAVRKKGYEHHR-
RnFV1-NW10      GMPLNGYWFDRVYSRHS-----RCQHHYGLDFKEFDSTLTAEVNKLTKAIRKKGFERHK-
ShTSA_fusa1     GLPLTGFHLSQLFEKHS-----KFAYHYGGDCTEFDSTITKNIQEIIKSIRKKGFSRHR-
GaMV2           GLPINGTNLSKIYAQHEITGGIDGATHFAGDCSDFDSTVTGPILDVIKHIRKRGFKDHR-
PPV             GMTKFRGGWDKLLKALP-----EGWIYCDADGSQFDSSLSPYLINAVLNIRLAFME----
                 :                            *    **

FgHV/HN10B      GNGANIASVAIAATRAKSSGWIWGLTEAE-HSALKVVIPEQGRRDTLVGYDSSRFIDATH
CHV1/EP713      GKGRQFAQVVQCKFEAMQNAWVMGITEPS-YTALTFHVPDVAVRHELESKYPAHFATFSE
CHV2/NB58       GKGAHFTKVIQCKYQAMQDAWVMGITEPT-YDNLVFHVPDNEARHELQKAYPKHFISFRE
ShTSA_hypo      -RGPEKASVLTAKYNALQDSFIFGLTMPNDFGAVSLCLPK-DVQDKLILSNPSKFIRLQD
SsHV2_5472      -----VLSWLKAKHEAMQNSYIAVLSLPR-------------------------------
CHV3/GH2        -QRDAIESAIDATYDNLVASWIIDIHS---------------------------------
SsHV1/Sz150     -QRAEIETAVDATYDNLIASWIIDIHT---------------------------------
VcHV1/MVC86     -QREAIETAIDATYDNLMASWIIDIHT---------------------------------
CHV4/SR2        -QRVAVESAIDATYDNLVASWIVDIHT---------------------------------
FgFV/DK21       -DHDRICNLIDVAYDQLEHQLLNTTST---------------------------------
RnFV1-NW10      -DYDRICALIDVNYDQVEHQLLHTTST---------------------------------
ShTSA_fusa1     -QYEIISTMIDINYERLNESKIVTPST---------------------------------
GaMV2           -SIKAISWLVDRNYDSIEKSLMVSSNT---------------------------------
PPV             -EWDIGEQMLSNLYTEIVYTPIAT--------------PD--------------------
                                     :

FgHV/HN10B      LSLAGVTELITSGQTL------------------GKLLLVASRNQANIPSSVKDLGSFIV
CHV1/EP713      LLAHNNVNVTEWKRLSWEERKACARDMQ---AVPGKVFLTNDPALRLQGSS---------
CHV2/NB58       LLDHNMTSLSTWNSLSGIDRIAYFKRFE---LPPGKVFLTTNPALRPARSS---------
ShTSA_hypo      IESSAPEMVSVAAKLMKGEISALNPSDEYHNLYKGKVVLVNSPFDSYVHQEVGDGKSRA-
SsHV2_5472      ----------------------------------GKEL---SPFLQHARAS---------
CHV3/GH2        ------------------------------------------------------------
SsHV1/Sz150     ------------------------------------------------------------
VcHV1/MVC86     ------------------------------------------------------------
CHV4/SR2        ------------------------------------------------------------
FgFV/DK21       ------------------------------------------------------------
RnFV1-NW10      ------------------------------------------------------------
ShTSA_fusa1     ------------------------------------------------------------
GaMV2           ------------------------------------------------------------
PPV             ------------------------------------------------------------
```

Figure S4. Multiple amino-acid sequence alignment of the ORF1 domains and ORF2 protein encoded by RnFV1-NW10 and their related virus and virus-like sequences. (A) RdRp domain, (B) Hel domain.

```
FgHV/HN10B      DNNANLMSTLRNKAHYSQTFIYHEGGFNIGTRQKGAKLVP-------RAMYDDLAKFADA
CHV1/EP713      ----------------------WQGSFTTEPKRDEFRKYQTYFYDSKAAMREDIKRIVFA
CHV2/NB58       ----------------------WQGSFTLEPKKDEYRKYQTYYCSSREAMKEDIKRIVFA
ShTSA_hypo      -RNLLFAPIYAEVIHGSVDRTNRESHFTTLERKDPYQ----------------FALDLFD
SsHV2_5472      -------------------------------NRDGPRALPGY------------------
CHV3/GH2        ------------------------------------------------------------
SsHV1/Sz150     ------------------------------------------------------------
VcHV1/MVC86     ------------------------------------------------------------
CHV4/SR2        ------------------------------------------------------------
FgFV/DK21       ------------------------------------------------------------
RnFV1-NW10      ------------------------------------------------------------
ShTSA_fusa1     ------------------------------------------------------------
GaMV2           ------------------------------------------------------------
PPV             ------------------------------------------------------------

FgHV/HN10B      PLALLSNMHYKNRGGDTGGNETTPYNTLSLRAIYRMAWSLTMNR-----PPKEFQTYN--
CHV1/EP713      NREVISNVHHKNRGGGTGQSATSWDNTATFKLGVISAWARATGK-----PPKDFFCSN--
CHV2/NB58       NRDVLSNIHHKNRGGGTGQSATSWDNTATFKLGVISAWARATGK-----RPADFFKTN--
ShTSA_hypo      HIDIAYNVFHKDKGGGTGENATSWDNSIGFRLSYYLGWWKYYDY---KISAEEVIQRH--
SsHV2_5472      -----ENWILKIRSGATGESSTAWTDSWTFRATFCLIVWDYCDYFSIPFSPDDFFNDSLM
CHV3/GH2        -----GRARFKRQGLSTGHATTTPSNTEYMRVLMLYSWKQITGR-----PYSEFYDCV--
SsHV1/Sz150     -----GRARLKKQGLSTGHATTTPSNTQYMRVLMLHAWKEITGR-----PYAEFYDTV--
VcHV1/MVC86     -----GRARFKRKGFSTGHATTTPTNTEYMRVLALYAWKKTTGR-----DYKDFYECV--
CHV4/SR2        -----GRARFKRQGLTTGHATTTSSNTDYMRALMLYAWKQVTGR-----TYDEFYQDV--
FgFV/DK21       -----GQVYKKGTGLTTGHSSTSADNSLGIAILYLFAWKELTGL-----SAREFVHFN--
RnFV1-NW10      -----GNIYQKGTGLTTGHSSTSMDNSIGTVVLYLMAWKKVTGL-----GAREFKHYN--
ShTSA_fusa1     -----GNVYNKGTGLTTGHASTSSDNSLTLISLYMAAWVNLTGL-----SAHEFRSFN--
GaMV2           -----GEVYRKGSGLMTGHASTSADNSLAMVAVYALAWRKLTHM-----KADDFLKYN--
PPV             -----GTIVKKFKGNNSGQPSTVVDNTLMVILAMTYSLLKL-GH-----HPDTHDCIC--
                          *  .  :*   *   ::

FgHV/HN10B      KMSNQGDDAMWSSFGKYGIRTYKQM---LKFKEICAQMGITMTIDST-----KDITKVEY
CHV1/EP713      RLYNTSDDTVWWS---KDLLSSAEV---DRFKQAAADFGILLEIGST-----KKITEVEY
CHV2/NB58       RFYNTSDDTVWWS---KDFLTSAEV---DRFKQAASDFGIMLEIGTT-----RKITEVEY
ShTSA_hypo      PLKNTGDDNIGSN---AIRRKDVDI---HRLQACFKEVGLNLEFTMV-----DNIEEMEY
SsHV2_5472      RLDNSGDDNMGHL---DFLKKYGHVLNPQVMVACAKNRNMTLDFAVL-----DSFEKCEF
CHV3/GH2        KFSSFSDDNFWST---NLDENVFSG---RLVSDFWLSRGVQVRVEGVS----DSLSDLSF
SsHV1/Sz150     KFSSFSDDNFWST---NLPPNVFSG---KLVSDFWLSKGVQVRVEGCS----DNLADLSF
VcHV1/MVC86     KFSSFSDDNFWST---SLAKSIFSG---KHIADFWLSRGVQVRVESES----DDLSKLSF
CHV4/SR2        KFTAFSDDNFWST---SLDPSVWSA---DKISEFWLSRGVQVRVEGAS----DDLSNLSF
FgFV/DK21       ELSDYGDDHVLSFL--ATKPAAWNF---KNIQKVMARWGVENRLEAS-----GPLDSIPF
RnFV1-NW10      ELSCFGDDHVLSFL--STRPVAWNI---SNIRSAMSSFGVSLKLDSE-----GSLEKIPF
ShTSA_fusa1     ELSCYGDDHILSFS--ENAPKVWTF---ENIQKIFKTWGITMKNEFPNADK-RDLEKISF
GaMV2           TLSVYGDDHILSIS--KTAPISWTF---PNIQAYLASCNITLREEVPTGGKGTKLESIPF
PPV             RYFVNGDDLVLAVH-----PAYESIY--DELQEHFSQLGLNYTFTTKT----ENKEELWF
                     .** .                    .       .:                .  :

FgHV/HN10B      LSKF--------------VRRPTP--QDSEDLAV-----WRRHKINEA
CHV1/EP713      LSKL--------------PRRPTA--EDSADYRA-----WRQGRIENM
CHV2/NB58       LSKV--------------PRQPTK--EDSEDYKA-----WRKGRLENL
ShTSA_hypo      LGKVYCPVDKY--TEGRSQRTPEAIKYDRATLDA-----WARSKFDMD
SsHV2_5472      LGCM--------------AREPTA--MDKKTLERVKAL-FHEAKVRPD
CHV3/GH2        LAKK--------------------FSFEQKHLDEVASLTGAHPKVAIV
SsHV1/Sz150     LSKK--------------------FSLDEKHLQEVKDITGAQPKVAIV
VcHV1/MVC86     LAKM--------------------FSFDEKHLAEIESLTGVKAKVAIV
CHV4/SR2        LAKR--------------------FSFDPHHLDEVRMHSGRDARVAIV
FgFV/DK21       LSKF--------------SRRLTA--EDRAVFAK--------YQVPLP
RnFV1-NW10      LSKW--------------IRKPTA--ADKADFKAAGLDNWSGYAVFHD
ShTSA_fusa1     LSKF--------------ARKSNH--TDDKLFKEVIGSSAPKYVIYHD
GaMV2           LSKM--------------ARKPNS--EEINHFTKLGIE-IPRWVISHD
PPV             MSHKGVLYDDMYIPKLEPERIVSILEWDRSNEP-----------IHRL
                :.                         :                .
```

B:Helicase domain

CLUSTAL format alignment by MAFFT (v7.146b)

```
FgHV/HN10       LAADTGTGKSSWW---LAALYGARRH--KNIRHVWVVSPYKSLRDNIDVPFGIK------
CHV1/EP713      VAAKTASGKSTFF---PAAVWAERRN--IGIKKLWIVMPRKILRDNWEIPFDIR------
CHV2/NB58       VAAKTASGKSTFF---PSAVWAERKN--LGIKKIWIVVPRIISGIFGTSPFDVP------
ShTSA_hypo      VSAGTGSGKSTLFNYSILSLSQTVWD--RPVKRVVVLMPRKILLKQWSSPLNVK------
SsHV2_5472      LSSETGTGKSSLG---VDAITRMIKHEIAPTSRTWVFVPTRILLKDPLPGFLQRGEDSDR
StTSA_hypo      LSSETGTGKSSLG---VDAITRMIKHEITPTSRTWVFVPTRILLKDPLPGFLQRGEDSDR
SsHV1/Sz150     ITSPPGAGKSTDF---ILSLK-------KEFETVIVACPRVILVQN-NPVAQTR------
CHV3/GH2        VTGVPGAGKSTDF---VISLK-------QKYETVIVACPRQILVKN-NPVAQTK------
VcHV1/          ITGPPGAGKSTDF---IQSLH-------SKYDTVIVACPRQILVRN-NPVAQSR------
CHV4/SR2        ITGPTGCGKSTDF---VVNLWR------GGYNTVIVACPRRILVRE-NPVAAVR------
FgV1/DK21       VQAGTGTGKSTSF---IKHLSLVVGA---RYNKIIVIEPRSALVRSLVPYVRDT------
RnFV1-NW10      VSAPTGSGKSTAL---VKCLAENVGH---LYEKLIVIEPRSLIVTSITPYTQTK------
CmESTc_fusa     VSAPTGSGKSTML---IHALATNVGF---MYEKIIVIEPRSAIVKSIVPYMQCS------
SiTSA_fusa2     IEAPTGSGKTTSF---IRHLLLTTGH---RFDKIIVVEPRSSIVSTVTPYVRTV------
SiTSA_fusa1     IEAPTGSGKTTSF---IRHLMMVSGS---RFDKIVVVEPRSAIVATVTPYVRTI------
ShTSA_fusa      IVAGTGTGKSTTM---ILYLQQSLGM---YFNKIIVIEPRSKVVKGLVSYVKT-------
ZtESTc_fusa     IQAPTGTGKSTAL---VHHVWETCKG---SHSRLVVVEPRTILIHGLVPYMRSF------
PPV             IRGAVGSGKSTGL---PFHLS--------KKGHVLLIEPTRPLAENVCKQLRGQ------
                : .  . **::        :               :  *

FgHV/HN10       ------TQVLMKGVQMN-NDFVKSATYGHFA--QARINQIDPERDVVLFDEFHLQT---M
CHV1/EP713      ------SQIVKRGKTLDPSADIYVTTYGHF---RTRIGGLVPRDNLVFFDEFHEMD---G
CHV2/NB58       ------SQKIRRGVTLNPNADIYVTTYGHF---LTRVPGLDLRENIVFFDEFHEMD---G
ShTSA_hypo      -GTHYPVQELSKGHTIDQNARILMGTYGHMLN-RI-NNNEFNENDVFFMDEFHELS---G
SsHV2_5472      NESLRTYQVLRKGIQIRSNAEILFMTYGHGRNRLM-SGEFQDGVDTAFVDEMHILS---A
StTSA_hypo      NESLRTYQVLRKGVQIRSNAEILFMTYGHGRNRLM-SGEFQDGVDTAFVDEMHILS---A
SsHV1/Sz150     -LYAGCEDDMTRGM-------INFGTAGYLRR----ILADLPPSTVICLDEFHEMD---E
CHV3/GH2        -LYSGCEDNLIRGY-------INFGTAGYLRR----TLADLPESTILCLDEFHEMD---E
VcHV1/          -LFAGCEDNLTQGY-------INFGTAGYLRR----ILADLPENTIIVLDEFHEMD---E
CHV4/SR2        -LWAGCPDVLTPGL-------INFGTAGYLRR----VLGELPDDALLVLDEFHELD---E
FgV1/DK21       --LLVDATGCTAGYDFDPTRKVWYMTPQEA---ILRHRHTFDRGNLIVVDECHLGEAAYR
RnFV1-NW10      --LGLDSSGATTGMTLDKSAKVWYVTAQEL---LL-HPSWVSPKNLIIMDECHVKEPPYM
CmESTc_fusa     --FALDCTGFTSGMVGDEQAKVIYCTASEF---LL-HSKWWNNSNLLVVDECHIDESAYL
SiTSA_fusa2     --LSLDATGCTSGMTMDPDAKVVYCTGQEL---LL-HQSWLSKNYLFVVDECHINEPAYE
SiTSA_fusa1     --LSLDATGCTSGMSMDPDAKVIYCTAQEL---LL-HASWLKKNYLFVVDECHISEPAYE
ShTSA_fusa      --IGVQASGLTTGLELDDREKVWYMTAQEL---LL-HPQWVSPSNLFVIDECHIDELPYK
ZtESTc_fusa     --LGLNCTGGTSGMEFDESCSVWYVTPTWF---FA-NTHRIKSDFIVILDEAHIVEPQYE
PPV             -PFNVNPTLRMRGMSTFGSTPITVMTSGYALHFLANNPTYLDNYKCIIFDECHVHDASAM
                            *        :   *                      .** *

FgHV/HN10       EIINALH---FNPA-RTFLLSAT------------PVDVPSLKNTPSLFPDIKRRFQPVV
CHV1/EP713      FMLQDVE---DWKG-PTIFMSAT------------PVALHGMAGIPFLEPTLPKRFNLTV
CHV2/NB58       FMLQGVE---KWKG-PTIFMSAT------------PVSLAGMEDIPFLEPSLPKRFPLTV
ShTSA_hypo      EMMACVQATYNTKI-RLFFLSAT------------PVTLPGIQTTFW-DAKFPPRFNKS-
SsHV2_5472      EQRLVVN---QLKGQRLIFSSATHVP---------PPGFTA----PVFRSTQTKKWKAIQ
StTSA_hypo      EQRLVVN---QLKGQRLIFSSATHVP---------PPGFTA----PVFRSTQTKKWKAIQ
SsHV1/Sz150     DTLWLLD---RYRG-QALTITAT------------PAFTGADRFVEV-RLSKGRNSRWT-
CHV3/GH2        DSLWLLD---RYRG-QCVVITAT------------PDFYGSQRFSEV-RLSKGRNSAWT-
VcHV1/          DTLWLLD---RYRE-HCIVVTAT------------PEFYGSNRFVEV-RLSKGRNSHWT-
CHV4/SR2        DTVWLWD---KYQG-QTIVMSAT------------PEFPGAERMTPV-ALQRSRSGGHV-
FgV1/DK21       VIQPFLK---SQSHLDSIYLTAT------------PSAFNFEQCEASVELNIANVWHVA-
RnFV1-NW10      MVRSIID---QGGT-NVLFLSAT------------IPSDVYDTCVTL-DLNTAKVWRVN-
CmESTc_fusa     LAGKVLM---DNMA-DQLWVSAT------------PTAHQVSAMHHI-PLNTARVWNLT-
SiTSA_fusa2     VTKTLLL---ASKV-HLILMSAT------------PNKADIDSCIHI-PLISARLYSIH-
SiTSA_fusa1     VVKTLLH---NASV-HLILMSATPLKADVALEGMLPLKADVDRCVHV-PLVSARLYSIH-
ShTSA_fusa      VVKEMLT---KTKGLTVLLATAT------------PTDEFSSICQTVTEIELPKIYRSYP
ZtESTc_fusa     AMKMYME---KFTS-YAIRVSAT------------LAEEDVKGHVLI-DIPIAQIWTVD-
PPV             AFRCLLSE-YSYPG-KILKVSAT------------PPGHEV-------EFKTQKEVKVIV
                                 .  :**

FgHV/HN10       KLYDDNMDVVDAYKEAEHLW---PHVMKKPQP-----RVLIVVPTIKQQSDTITLLQDLL
CHV1/EP713      -YKVDSDDVLEMWNRARNQF--------ADQPE-LLARPMIIVPTYNELKKTIAGLENLD
CHV2/NB58       -YKVDSDDVLEMWNRARNQF--------ADEPD-ILARPMVIVPTYKEVKKTIAGLENLD
ShTSA_hypo      -IYIRDDTPVNNFFWAQKQF-----------PE-HAKSAIIRLSTFAEVEQVREALSYRN
SsHV2_5472      RIFPATTNVASMFQRAKNDT--EPMAGMKEAPAQLSDRTLILCSTFRELDEVAESLTTLR
```

```
StTSA_hypo      RIFPATTNVASMFQRARNDT--EPMAGMKEAPAQLSDRTLILCSTFRELDEVAESLTTLR
SsHV1/Sz150     -VHDEIRKGVPKLTDAWDEL-------MLYHES--NERVLVILPTVNDVETCVRHAQQLA
CHV3/GH2        -IQDDFRDTPGKLEDGWNCL-------MESAKT--NDRVLMIVPSIQDVETCKRHAAQLV
VcHV1/          -VMDDLRSGKGNLEEAWNEL-------ITHTND--DKKILMIVPTVKDVKTTLHHIEQLV
CHV4/SR2        -TTTYIKDTAGKLQDAWDEL-------LAPSPT--PGPTLVVLPTVADVEWMAHHALALA
FgV1/DK21       --VDHPVSRAAEVGAYLKDYRALVLDCLRGLPR--ISKVLVFYPSKEGAISFADSI---D
RnFV1-NW10      -TMHHTFEDLVSARSVLDHYAAEAVSIVMKLPA--NAKVLIFSQTVAEATSIAERV---G
CmESTc_fusa     -TTRSTNKLVGTYKDAFNSYCHQVMSAINNLPK--SSKILVFVNTIKEGLTVCDQV---G
SiTSA_fusa2     -ESRESMNSVRSEQQFNEAYMQMVGSIIQSKPK--MSKALVFCTTTGMAYQALSRY---S
SiTSA_fusa1     -ESRESLSGVRSLEQFNEGYLQMVGSIVSAKPK--MSKALVFCTTTGMAYQAMSRY---A
ShTSA_fusa      KEYANNVEIPIKGSSWVHRYLGIVTEIMSNYRT--PEKFLIFINDKRDLDIFRQNL---R
ZtESTc_fusa     -DSIEKQNS----DSPTSHYKKWAASYVNALPA--GVRAAVIVDTPEDARSLRERC---I
PPV             EESLSFQQFVSNLGTGCNS------DILKH-----GVNVLVYVASYNEVDTLSKLL---T
                                                        :

FgHV/HN10       PNGT------LINPYSRLHRNEPP-----------------EGIIVSTPYVDVGTNFKN-
CHV1/EP713      RSIT-------WHEVSSNSPLVPK-----------------TGGLVCTPYVQTGIDIKP-
CHV2/NB58       RNLR-------WQEVSRKNPKVPM-----------------TGGMVCTPYVQTGIDIKP-
ShTSA_hypo      IRTF------EVSSATADQEIPSD-----------------DTVLVTTQVIDVGMNL-P-
SsHV2_5472      KSMFGGGIGVSLPPVVEISSRVKP--GTEAWTQRQTAFVKGRYIALGTKQAATGMDIKPF
StTSA_hypo      KSMFGGGIGVSLPPVVEISSRVKP--GTEAWSQRQNAFIKGRYIALGTKQAATGMDIKPF
SsHV1/Sz150     KGKR-------IRKLYRGTNTVVE-----------------ADWYFATSIVDAGITI-P-
CHV3/GH2        TNKR-------VCGLYRGQNTVTE-----------------ADWYFATSIVDAGLTI-P-
VcHV1/          PGKR-------ACGLYRGNTMVRP-----------------ADWYVATSVVDAGLTI-P-
CHV4/SR2        PGKR-------FCVLHRGRDTVTE-----------------ADWYFATSIVDAGLTI-P-
FgV1/DK21       RPVS------FLNSGSHDTTG--------------------SVILSTSVADAGLTI-P-
RnFV1-NW10      RRAF------VLSG-SSKTGDMSG-----------------YNVIVCTSVADTGITI-P-
CmESTc_fusa     QRAR------LLSS-SSDIDSISG-----------------GSVIVCTSVADVGLTL-P-
SiTSA_fusa2     GRGC------VLSS-TSKVDNIDE-----------------FDTIFTTSVADVGLTL-P-
SiTSA_fusa1     GRAC------VLSS-TSKVDNLSD-----------------FDTIFTTSVADVGLTL-P-
ShTSA_fusa      GRGF------FLSSETEDVDLSEE-----------------VDFIVTTAVCDVAITI-P-
ZtESTc_fusa     RDCQ------CLSS--QEDPVVMP-----------------HSVYAATSVVDVGITL-P-
PPV             DRSF------KVSKVDGRTMKVGNVEIPTSGTQA------KPHFVVATNIIENGVTL---
                                                               *     .  :

FgHV/HN10       PPDVLIDAGKQVLID------RGKMI--LPLPWTDPDTDKQRQGRVAR-----KG
CHV1/EP713      APSILIDSGRDVIVH------KGRLV--TPHPYTDEKTNEQRVNRVGRTM---DG
CHV2/NB58       APTILIDSGRDVVIH------KGRMV--HPHPYTDDKTNEQRINRVGRIM---KG
ShTSA_hypo      GRRLMIPSGKMIKNV------RGQM----EMGWTDRDTEHQIAGRVGRFQ---ND
SsHV2_5472      PPRLLIDGGEDIYSH------QGSIL---KLPTTQRDHE-QRIGRVTRNSATGDG
StTSA_hypo      PPRLLIDGGEDIYSH------QGSIL---KLPTTQRDHE-QRIGRVTRNSATGDG
SsHV1/Sz150     HVGVVIDMGYSLGYS------KGKF----TKRPSSKNISVQRKGRTGRTC---NG
CHV3/GH2        GLTKIIDLGWSSGYK------HGKF----IKRPSSRNISAQRRGRTGRTC---AG
VcHV1/          DVSIVIDTGWSLGFK------GGKF----QRRPSSRNISVQRRGRTGRTT---NG
CHV4/SR2        GLTRVIDTGWSSGWS------QGKF----RRRPSSHNVADQRRGRTGRTC---DG
FgV1/DK21       AVDLVVSPCLDYTTTGFG---LEVT----YALLNQMQIK-QRQGRTGRTN---NG
RnFV1-NW10       DVDTIITSDVGFVVEHGVKE-SSKL----YYRLSADDLK-QRVGRTGRTN---HG
CmESTc_fusa      EVDTVMSMDIGFTVLESLES-SKPV----YFRLNSSHIS-QRAGRTGRTN---HG
SiTSA_fusa2      DIDLVITSDIGFTVESLMSS-SRKI----YYKLPPPAIK-QRVGRTGRTN---NG
SiTSA_fusa1      DIDLVVTPDIGFTVESLLSK-ANKI----YFKLPPSAIK-QRVGRTGRTN---NG
ShTSA_fusa       GVTVVITPNFTRKVSYDRNGLSKPC----FALLDSATLK-QRSGRTGRTN---NG
ZtESTc_fusa      DLDVIISPDWVY---------DGKS----RVALNDTTRR-QRRGRVGRVR---NG
PPV              DIDVVVDFGLKVVPVLDI---DNRLVRYTKKSISYGERI-QRLGRVGRNK---PG
                      ::                                    *   .*. *      .
```
